# Effects of Visual Art Therapy on Positive Symptoms, Negative Symptoms, and Emotions in Individuals with Schizophrenia: A Systematic Review and Meta-Analysis

**DOI:** 10.3390/healthcare12111156

**Published:** 2024-06-06

**Authors:** Shih-Cing Du, Chih-Yen Li, Ya-Yun Lo, Yu-Hsuan Hu, Chi-Wei Hsu, Chung-Yin Cheng, Tzu-Ting Chen, Pei-Hsuan Hung, Pao-Yen Lin, Chyi-Rong Chen

**Affiliations:** 1 Department of Occupational Therapy and Graduate Institute of Behavioral Science, College of Medicine, Chang Gung University, Taoyuan 33302, Taiwan; b0906013@cgu.edu.tw; 2 Department of Occupational Therapy, Shu-Zen Junior College of Medicine and Management, Kaohsiung 821004, Taiwan; s51110077@student.szmc.edu.tw (C.-Y.L.); s51110017@student.szmc.edu.tw (Y.-Y.L.); s51110020@student.szmc.edu.tw (Y.-H.H.); tracy5824@cgmh.org.tw (T.-T.C.); phh@ms.szmc.edu.tw (P.-H.H.); 3 Department of Psychiatry, Kaohsiung Chang Gung Memorial Hospital, College of Medicine, Chang Gung University, Kaohsiung 833401, Taiwan; harwic@cgmh.org.tw (C.-W.H.); alto1830@cgmh.org.tw (C.-Y.C.); py1029@cgmh.org.tw (P.-Y.L.)

**Keywords:** schizophrenia, visual art therapy, meta-analysis, psychiatric symptoms, emotions

## Abstract

Schizophrenia is characterized by psychiatric symptoms and emotional issues. While pharmacological treatments have limitations, non-pharmacological interventions are essential. Art therapy has the potential to enhance emotional expression, communication, and health; however, the effectiveness of visual art therapy remains uncertain. This systematic review and meta-analysis synthesizes the findings of randomized controlled trials (RCTs) of visual art therapy on positive symptoms, negative symptoms, and emotions in patients with schizophrenia. This study reviews RCTs published prior to February, 2024. The PubMed, Embase, Cochrane Library, CEPS, CNKI, Wanfang, and Yiigle databases were searched, and three independent researchers screened the studies. In this meta-analysis, standardized mean difference (SMD) was employed as a measure to calculate effect sizes for continuous variables using a random effects model, while the meta-regression and subgroup analyses were performed with patient and intervention characteristics. A total of 31 studies revealed visual art therapy had a significant small-to-moderate effect on positive symptoms (SMD = 0.407, 95% CI 0.233 to 0.581), a moderate effect on negative symptoms (SMD = 0.697, 95% CI 0.514 to 0.880), a moderate effect on depression (SMD = 0.610, 95% CI 0.398 to 0.821), and a large effect on anxiety (SMD = 0.909, 95% CI 0.386 to 1.433). The subgroup analysis revealed painting and handcrafts had significant effects on positive symptoms, negative symptoms, and emotions. Combined Chinese calligraphy and painting had significant effects on positive symptoms, depression, and anxiety. Better improvement was noted among the Asian population, and a longer weekly treatment duration was associated with better improvement in positive symptoms. Female participants tended to have more improvements in negative symptoms and anxiety through visual art therapy. The results indicate that visual art therapy has positive effects on the psychiatric symptoms and emotions of individuals with schizophrenia. We recommend future research further investigate art therapy modalities and durations.

## 1. Introduction

Schizophrenia is a psychiatric disorder characterized by positive symptoms, including delusions and hallucinations, negative symptoms, such as apathy and avolition, and general psychopathology (e.g., depression and anxiety) [1,2,3], affecting approximately 1% of the population during their lifetime [4]. Individuals with schizophrenia often experience challenges related to unemployment, social isolation, loneliness, and unstable housing. Numerous individuals diagnosed with schizophrenia continue to experience symptoms despite receiving pharmacotherapy, and 10 to 50% of patients exhibit suicidal tendencies [5]. Both the psychiatric symptoms and emotional issues associated with schizophrenia require proactive intervention.

Pharmacotherapy has demonstrated significant efficacy in addressing the positive symptoms, as well as the symptoms of depression and anxiety associated with schizophrenia [6,7,8]; however, its effectiveness in alleviating negative symptoms remains limited [9]. Furthermore, pharmacotherapy often comes with side effects, including restlessness, sedation and sexual dysfunction, and poor treatment adherence [10,11]. Therefore, adjunctive non-pharmacological therapies are frequently required to enhance outcomes, among which cognitive behavioral therapy and psychosocial interventions hold promise [12,13]. Meanwhile, art therapy represents an alternative non-verbal form of expression that has been widely utilized in the treatment of mental disorders [14]. Previous studies suggest that art therapy may entail fewer side effects [15], thus warranting further exploration.

The British Association of Art Therapists defines art therapy as a process of expressing thoughts and emotions that have not been expressed through artwork and interaction with a therapist [16]. Previous studies have categorized art therapy into various types, including visual art therapy, music therapy, dance and movement therapy, drama therapy, and expressive and writing therapy [17]. In a later study, an extensive literature review published by Joschko et al. (2022) categorized art therapy into five categories, combined with other conventional therapies, including the visual arts, music, performing arts, architecture, and literature [18]. Among these categories, visual art therapy using drawings and paintings has been recognized as the most beneficial form of therapeutic process within the fields of psychiatry and psychology [14]. Furthermore, other art therapy forms have gradually become established within various professions, including music therapy, dance and movement therapy, and drama therapy [19,20]. The literature indicates that different diagnosed mental illnesses may elicit varying responses to different art therapy modalities [17,21]. Therefore, it is necessary to investigate the differential effects of visual art therapy on patients with schizophrenia.

The mechanisms underlying the effects associated with visual art therapy remain unclear. In general, art serves as a bridge between individuals with mental disorders and therapists, offering a secure and indirect avenue for self-expression and interpersonal connection [22]. Art therapy assists patients in acquiring artistic abilities, thereby enhancing their confidence, self-esteem, coping mechanisms, mood regulation, and social integration, while mitigating symptoms associated with mental disorders [17,21]. Additionally, it can facilitate conversation within the inpatient therapeutic environment, creating a more comfortable or liberating space while enhancing levels of trust in healthcare professionals and their treatment strategies [23,24]. Furthermore, art therapy commonly occurs in group settings, where art acts as a communal means of self-expression, enhancing an individual’s sense of belonging within a group environment [25].

In recent years, research has begun to explore the efficacy of art therapy in addressing symptoms of mental disorders. One study revealed a steady increase in the quantity of research on art therapy over the past 15 years, with painting accounting for 84% of the studies [20]. A previous review indicated that art therapy was considered meaningful and beneficial by the therapist and patients with psychosis; however, the effects of art therapy in symptom reduction were limited [22]. Additionally, two meta-analyses incorporated RCTs, with the results of both studies indicating a non-significant effect on the positive symptoms of schizophrenia, although there was a small-to-moderate effect on negative symptoms (standardized mean difference (SMD) = −0.39; *g* = −0.42) [26,27]. Of note, both of these meta-analyses included only nine studies and encompassed various forms of art therapy, such as music and dance; thus, they could not provide evidence regarding the efficacy of visual art therapy for schizophrenia. Meanwhile, another meta-analysis focused on the efficacy of therapy using Chinese calligraphy on neuropsychiatric symptoms, with the results indicating a significant therapeutic effect [28]. Although this report encompassed various participant groups, such as students and individuals with mild cognitive impairments [28], it still exhibited considerable heterogeneity in terms of art therapy modalities and study populations, thereby failing to provide direct evidence regarding the benefits of visual art therapy for individuals with schizophrenia.

Despite the potential benefits of visual art therapy, existing meta-analyses do not comprehensively examine the effectiveness of visual art therapy in patients with schizophrenia. Therefore, the aims of this study were as follows: (1) to systematically review RCTs of visual art therapy to determine the effects on symptoms and emotions in individuals with schizophrenia; (2) to identify moderators of visual art therapy via meta-regression, including sample characteristics and intervention variables; and (3) to conduct subgroup analyses to investigate the effectiveness of different types of visual art therapy activities.

## 2. Materials and Methods

### 2.1. Search Strategy

This systematic review adhered to the guidelines outlined in the Preferred Reporting Items for Systematic Reviews and Meta-Analyses (PRISMA) [29]. Three researchers (C.-Y.L., Y.-Y.L., and Y.-H.H.) independently conducted literature searches in the following databases: PubMed, Embase, Cochrane Library, and Chinese databases such as the Airiti Library (CEPS), China National Knowledge Infrastructure (CNKI), Wanfang Database, and Yiigle. Concurrently, ongoing clinical trials were searched on platforms such as clinicaltrials.gov and the China Clinical Trial Registry (ChiCTR). The literature search was performed from inception to the end of February 2024.

The search utilized a combination of keywords and appropriate Boolean logic, including terms such as schizophrenia, severe mental illness, art therapy, drawing, sketching, craft, calligraphic, painting, coloring, sandpainting, collage making, artistic pottery, and sculpting. The corresponding Chinese keywords were also utilized for the search. Details of the search strings and the database used are listed in Table S1. Additionally, manual searches were conducted for references cited in the included literature.

### 2.2. Inclusion and Exclusion Criteria

The inclusion criteria for literature selection were as follows: (1) adoption of an RCT research design; (2) inclusion of adult individuals diagnosed with schizophrenia as study participants; (3) intervention involving therapeutic activities such as drawing, handcrafts, calligraphy, or other forms of visual art therapy; and (4) assessment of outcomes including positive symptoms, negative symptoms, and emotional evaluation. Exclusion criteria encompassed the following: (1) utilization of art therapy types such as music, dance and movement, drama or theater, and creative writing; and (2) artistic product manipulation as part of pre-vocational training. Additionally, there were no language restrictions on the included literature. The literature selection process involved independent screenings by three researchers (C.-Y.L., Y.-Y.L., and Y.-H.H.), and any discrepancies were resolved through discussion with the corresponding author to reach a consensus.

### 2.3. Data Extraction

Two independent researchers (T.-T.C. and C.-Y.C.) employed a pre-designed data extraction form to gather study information, such as author names, publication years, countries, demographic information (e.g., participant age, gender), and study design details (e.g., intervention frequency, duration). Any discrepancies were resolved through discussion to reach a consensus. This study extracted data related to positive symptoms, negative symptoms, depression, and anxiety in individuals with schizophrenia.

### 2.4. Quality Assessment of the Literature

This study employed the Cochrane Risk of Bias (RoB) tool to assess the quality of the literature, covering aspects including selection bias, allocation concealment, performance bias, detection bias, attrition bias, publication bias, and other potential biases [30]. We used the Revman 5.4 software to generate the risk of bias graph and summary table. The Revman 5.4 software was developed by the Cochrane Collaboration and is primarily used for conducting systematic reviews and meta-analyses. It supports 16 languages, including English and Chinese. The assessment was independently conducted by two researchers (T.-T.C. and C.-Y.C.) within the research team. In cases of disagreement, consensus was reached through discussion with the corresponding author.

### 2.5. Meta-Analysis

The meta-analysis was conducted using the average change scores and standard deviations between the pre-test and post-test values [30]. Given the potential heterogeneity among various studies, a random effects model was employed to calculate the SMD and 95% confidence intervals (95% CI) for each outcome. The SMD is also known as Cohen’s *d* [31]. According to Cohen’s guidelines, effect sizes of 0.2 are considered small, 0.5 moderate, and 0.8 large [32]. Subgroup analyses were performed based on the different visual art therapy modalities employed (e.g., handcrafts, painting, calligraphy, etc.), publication years (within the last 10 years or over 10 years ago), and the continents where the studies were conducted. Meta-regressions were carried out based on participant characteristics, including age, gender ratio, duration of illness, and intervention characteristics, including weekly treatment time. Sensitivity analysis involved systematically excluding one study at a time to assess the impact of each included piece of literature on the meta-analysis results. The study utilized *I*^2^ for assessing heterogeneity, where *I^2^* values of 25%, 50%, and 75% represented low, moderate, and high heterogeneity, respectively. Publication bias was assessed by visual inspection of the funnel plot and Egger’s test. In addition, we conducted a trim-and-fill method for the adjusted analysis. All statistical procedures were conducted using Comprehensive Meta-Analysis Version 4 software (Biostat Inc., Englewood, NJ, USA). This study is registered in the INPLASY (INPLASY202430021).

### 2.6. Certainty of Evidence

We assessed the quality of evidence using the Grading of Recommendations, Assessment, Development, and Evaluations (GRADE) metric [33]. The GRADE approach evaluated the certainty of evidence in RCTs by considering risk of bias, inconsistency, indirectness, imprecision, and publication bias. We used the GRADEpro software to generate the evidence table [34].

## 3. Results

### 3.1. Search Results and Study Characteristics

**Figure 1:**
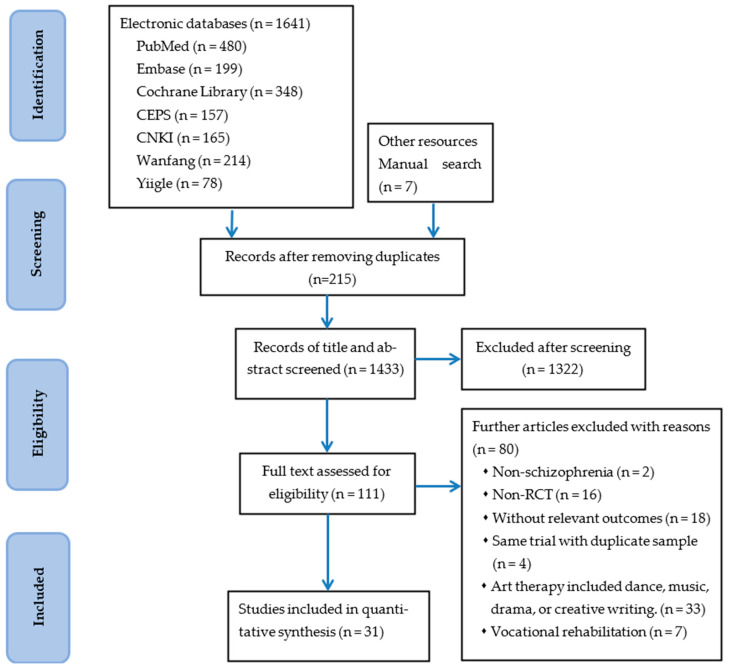
PRISMA flow diagram.

A total of 1641 records were identified across seven databases and manual searches. After removing 215 duplicate articles, we screened the titles and abstracts of 1433 articles. Of these, 1322 articles did not meet the inclusion criteria and were excluded. A full-text review was conducted on 111 articles, with 80 articles excluded based on the specified criteria. The reasons for exclusion and detailed information on the search results are summarized in Figure 1.

A total of 31 studies meeting the criteria were included for further qualitative and quantitative analysis (Figure 1). The characteristics and demographic data of the 31 studies are presented in Table S2. The studies were published between 2005 and 2023, with sample sizes ranging from 22 to 140. Two studies were conducted in the United Kingdom [35,36], two in Taiwan [37,38], one each in Germany [39] and Turkey [40], and the remaining twenty-five in China [41,42,43,44,45,46,47,48,49,50,51,52,53,54,55,56,57,58,59,60,61,62,63,64,65]. A total of 3037 individuals with schizophrenia participated in these studies. Of these, 1520 were allocated to the experimental group receiving art therapy, while 1517 were assigned to the control group receiving conventional treatment or usual treatment. The average age of participants was 38.8 years (range = 27.5 to 48.4 years), with an average duration of illness of 10.6 years (range = 2.72 to 29.5 years), and 51% were male. As for the setting, three studies were conducted in community mental health settings [36,40,56], and one study recruited participants from both community and hospital settings [35]. One study was conducted in a forensic mental health setting [52], and the remaining twenty-six studies were conducted in hospital settings. In terms of visual art therapy modalities, nineteen studies primarily employed painting [35,36,37,39,40,42,43,44,45,47,48,51,52,53,54,56,57,58,64], six studies utilized handcrafts [46,49,50,61,62,63], two studies solely focused on calligraphy [38,65], and four studies combined painting with calligraphy [41,58,59,60].

### 3.2. Risk of Bias in Individual Studies

**Figure 2:**
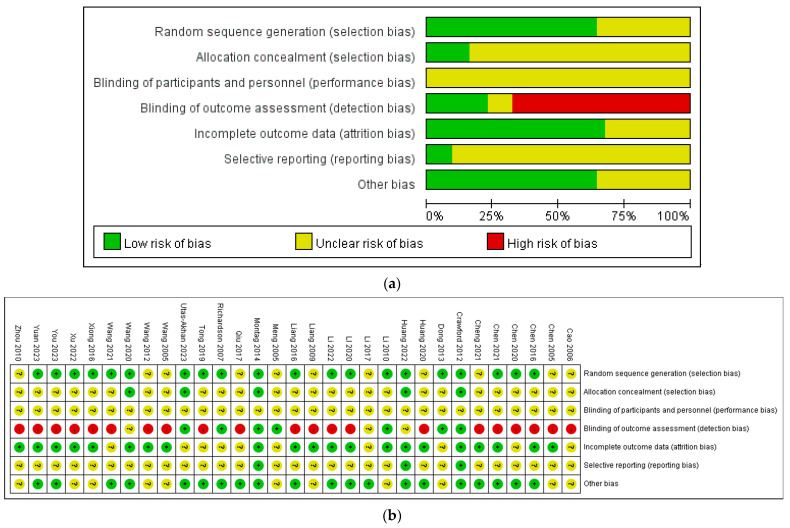
(**a**) Risk of bias graph. (**b**) Risk of bias summary for the included studies. Cao 2006 is the Ref. [65]; Chen 2005 is the Ref. [63]; Chen 2016 is the Ref. [37]; Chen 2020 is the Ref. [64]; Chen 2021 is the Ref. [62]; Cheng 2021 is the Ref. [61]; Crawford 2012 is the Ref. [35]; Dong 2013 is the Ref. [60]; Huang 2020 is the Ref. [59]; Huang 2022 is the Ref. [38]; Li 2010 is the Ref. [55]; Li 2017 is the Ref. [56]; Li 2020 is the Ref. [58]; Li 2022 is the Ref. [57]; Liang 2009 is the Ref. [46]; Liang 2016 is the Ref. [54]; Meng 2005 is the Ref. [53]; Montag 2014 is the Ref. [39]; Qiu 2017 is the Ref. [52]; Richardson 2007 is the Ref. [36]; Tong 2019 is the Ref. [51]; Utas-Akhan 2023 is the Ref. [40]; Wang 2005 is the Ref. [50]; Wang 2012 is the Ref. [49]; Wang 2020 is the Ref. [47]; Wang 2021 is the Ref. [48]; Xiong 2016 is the Ref. [45]; Xu 2022 is the Ref. [44]; You 2023 is the Ref. [43]; Yuan 2023 is the Ref. [42]; Zhou 2010 is the Ref. [41].

The risk of bias assessment is presented in Figure 2. Eleven studies were rated as unclear due to an insufficient description of the random allocation method, while another twenty studies were rated as low risk. For allocation concealment, 26 studies were rated as unclear, and 5 studies were rated as low risk. Regarding blind assessment, 21 studies were evaluated as high risk, 3 studies as unclear, and 7 studies provided detailed descriptions of assessor blinding, resulting in a rating of low risk. In terms of incomplete outcome reporting, 10 studies lacked clear explanations regarding participant withdrawals and were rated as unclear, while 21 studies were rated as low risk. For selective reporting, 28 clinical trials did not have trial registration, resulting in an unclear rating, while 3 studies were rated as low risk. Lastly, in the category of other bias, 11 studies were rated as unclear, and 20 studies were rated as low risk.

### 3.3. Synthesis of Results

**Figure 3:**
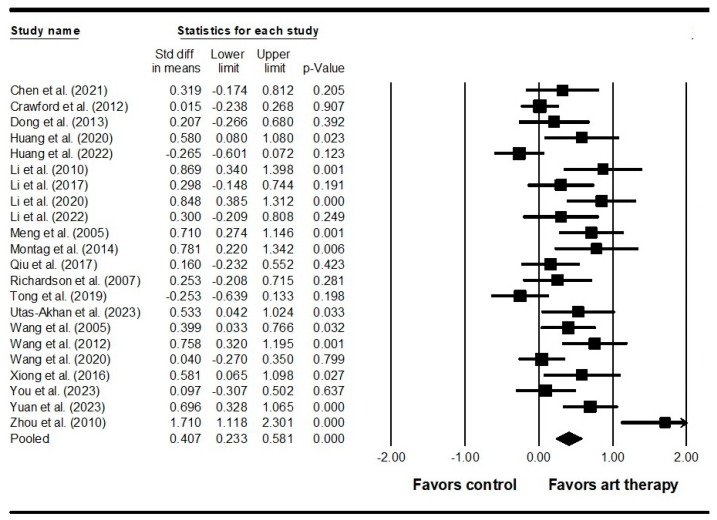
Forest plot of visual art therapy effects on positive symptoms in individuals with schizophrenia. Chen 2021 is the Ref. [62]; Crawford 2012 is the Ref. [35]; Dong 2013 is the Ref. [60]; Huang 2020 is the Ref. [59]; Huang 2022 is the Ref. [38]; Li 2010 is the Ref. [55]; Li 2017 is the Ref. [56]; Li 2020 is the Ref. [58]; Li 2022 is the Ref. [57]; Meng 2005 is the Ref. [53]; Montag 2014 is the Ref. [39]; Qiu 2017 is the Ref. [52]; Richardson 2007 is the Ref. [36]; Tong 2019 is the Ref. [51]; Utas-Akhan 2023 is the Ref. [40]; Wang 2005 is the Ref. [50]; Wang 2012 is the Ref. [49]; Wang 2020 is the Ref. [47]; Xiong 2016 is the Ref. [45]; Xu 2022 is the Ref. [44]; You 2023 is the Ref. [43]; Yuan 2023 is the Ref. [42]; Zhou 2010 is the Ref. [41].

**Figure 4:**
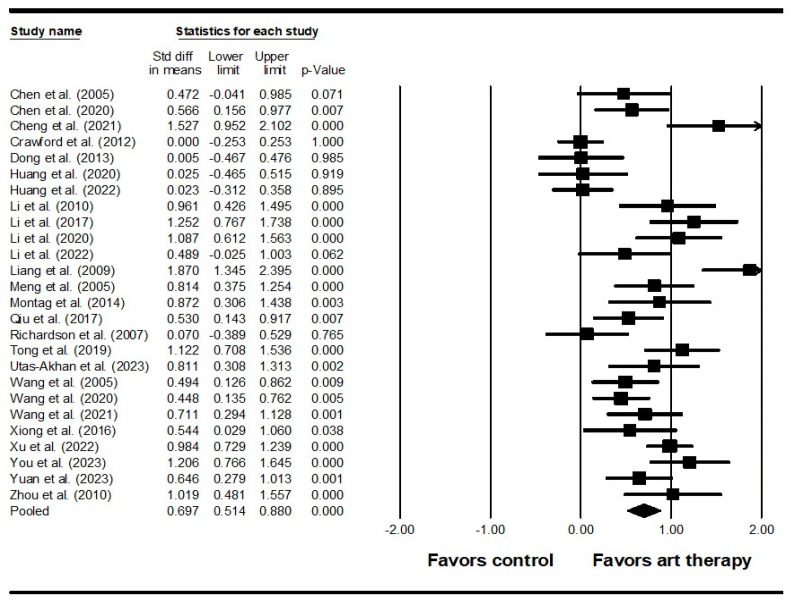
Forest plot of visual art therapy effects on negative symptoms in individuals with schizophrenia. Chen 2005 is the Ref. [63]; Chen 2020 is the Ref. [64]; Cheng 2021 is the Ref. [61]; Crawford 2012 is the Ref. [35]; Dong 2013 is the Ref. [60]; Huang 2020 is the Ref. [59]; Huang 2022 is the Ref. [38]; Li 2010 is the Ref. [55]; Li 2017 is the Ref. [56]; Li 2020 is the Ref. [58]; Li 2022 is the Ref. [57]; Liang 2009 is the Ref. [46]; Meng 2005 is the Ref. [53]; Montag 2014 is the Ref. [39]; Qiu 2017 is the Ref. [52]; Richardson 2007 is the Ref. [36]; Tong 2019 is the Ref. [51]; Utas-Akhan 2023 is the Ref. [40]; Wang 2005 is the Ref. [50]; Wang 2020 is the Ref. [47]; Wang 2021 is the Ref. [48]; Xiong 2016 is the Ref. [45]; Xu 2022 is the Ref. [44]; You 2023 is the Ref. [43]; Yuan 2023 is the Ref. [42]; Zhou 2010 is the Ref. [41].

**Figure 5:**
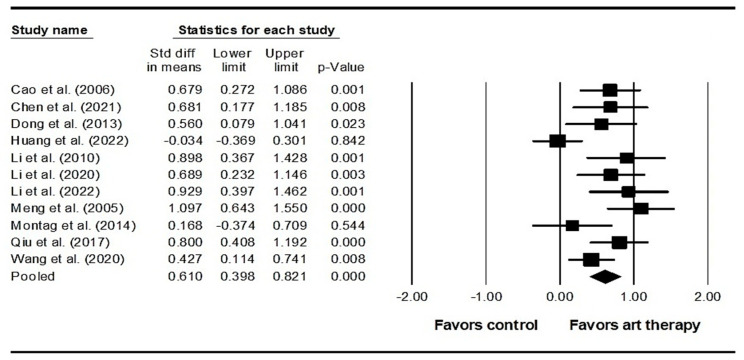
Forest plot of visual art therapy effects on depression in individuals with schizophrenia. Cao 2006 is the Ref. [65]; Chen 2021 is the Ref. [62]; Dong 2013 is the Ref. [60]; Huang 2022 is the Ref. [38]; Li 2010 is the Ref. [55]; Li 2020 is the Ref. [58]; Li 2022 is the Ref. [57]; Meng 2005 is the Ref. [53]; Montag 2014 is the Ref. [39]; Qiu 2017 is the Ref. [52]; Wang 2020 is the Ref. [47].

**Figure 6:**
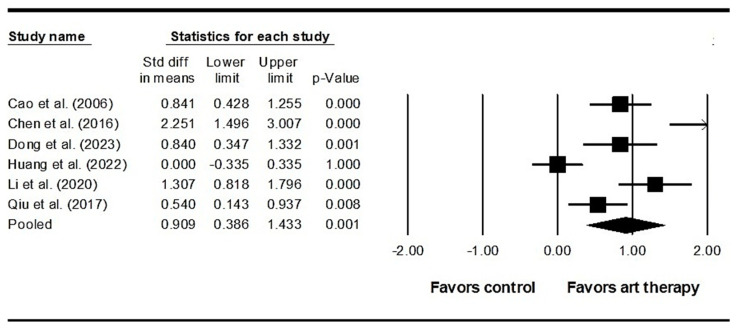
Forest plot of visual art therapy effects on anxiety in individuals with schizophrenia. Cao 2006 is the Ref. [65]; Chen 2016 is the Ref. [37]; Dong 2013 is the Ref. [60]; Huang 2022 is the Ref. [38]; Li 2020 is the Ref. [58]; Qiu 2017 is the Ref. [52].

Across 22 studies enrolling 2033 participants, the results indicated that visual art therapy had a significant effect on positive symptoms (SMD = 0.407, 95% CI = 0.233 to 0.581, *p* < 0.001) (Figure 3). Additionally, the Egger’s test indicated publication bias (*p* < 0.001). Therefore, the effect size was recalculated using the Duval and Tweedie trim-and-fill method, with four studies being adjusted, and the adjusted SMD was 0.287 (95% CI = 0.105 to 0.470). Across 26 studies enrolling 2539 participants, it was found that visual art therapy also had a significant effect on negative symptoms (SMD = 0.697, 95% CI = 0.514 to 0.880, *p* < 0.001) (Figure 4). Publication bias was not significant using Egger’s test (*p* = 0.058). There was significant heterogeneity among the included studies in terms of positive symptoms and negative symptoms (Q = 77.689, *I^2^* = 72.969, *p* < 0.001 and Q = 123.222, *I^2^* = 79.711, *p* < 0.001, respectively). As for emotional outcomes, a significant difference with heterogeneity was detected in depression (SMD = 0.610, 95% CI = 0.398 to 0.821, *p* < 0.001; Q = 25.848, *I^2^* = 61.313, *p* = 0.004) (Figure 5) in 11 studies enrolling 973 participants. No publication bias was detected (Egger’s test *p* = 0.113). In 6 studies enrolling 527 participants, the results revealed that visual art therapy had a significant effect on anxiety with heterogeneity (SMD = 0.909, 95% CI = 0.386 to 1.433, *p* < 0.001; Q = 40.240, *I^2^* = 87.575, *p* < 0.001) (Figure 6). The Egger’s test indicated publication bias (*p* = 0.005). In addition, the effect size was unchanged after adjustment by the Duval and Tweedie trim-and-fill method.

The results of the meta-analysis are summarized in Table 1, while the funnel plots of each comparison are presented in Figures S1–S4. The sensitivity analysis showed no change in the overall results after individually removing the included studies one at a time.

### 3.4. Subgroup Analyses and Meta-Regression

The subgroup analysis based on different modalities of visual art therapy revealed that painting resulted in significant improvements in positive symptoms (SMD = 0.333, 95% CI = 0.153 to 0.513, *p* < 0.001), negative symptoms (SMD = 0.704, 95% CI = 0.510 to 0.897, *p* < 0.001), depression (SMD = 0.681, 95% CI = 0.425 to 0.938, *p* < 0.001), and anxiety (SMD = 1.317, 95% CI = 0.428 to 2.207, *p* = 0.004). In addition, handcrafts had significant effects on positive symptoms (SMD = 0.492, 95% CI = 0.239 to 0.744, *p* < 0.001), negative symptoms (SMD = 1.076, 95% CI = 0.374 to 1.777, *p* = 0.003), and depression (SMD = 0.681, 95% CI = 0.177 to 1.185, *p* = 0.008). No individual study was identified among the current research investigating the effects of handcrafts on anxiety. The results revealed non-significant effects of calligraphy on positive symptoms (SMD = −0.265, 95% CI = −0.601 to 0.072, *p* = 0.123), negative symptoms (SMD = 0.023, 95% CI = −0.312 to 0.358, *p* = 0.895), depression (SMD = 0.313, 95% CI = −0.386 to 1.011, *p* = 0.380), and anxiety (SMD = 0.412, 95% CI = −0.413 to 1.236, *p* = 0.328). When combined, painting and calligraphy showed significant effects on positive symptoms (SMD = 0.824, 95% CI = 0.225 to 1.422, *p* = 0.007), depression (SMD = 0.712, 95% CI = −0.356 to 1.069, *p* < 0.001), and anxiety (SMD = 0.840, 95% CI = 0.347 to 1.332, *p* = 0.001); however, no significant effect was found on negative symptoms (SMD = 0.491, 95% CI = −0.059 to 1.042, *p* = 0.080). The results of the meta-analysis are summarized in Table 1, while the forest plots of each comparison are presented in Figures S5–S19.

The subgroup analysis based on publication years indicated that visual art therapy resulted in significant improvements in positive symptoms (SMD = 0.578, 95% CI = 0.272 to 0.883, *p* < 0.001), negative symptoms (SMD = 0.615, 95% CI = 0.231 to 0.999, *p* = 0.002), depression (SMD = 0.801, 95% CI = 0.568 to 1.035, *p* < 0.001), and anxiety (SMD = 0.841, 95% CI = 0.428 to 1.255, *p* < 0.001) in the studies conducted before 2014. There were also significant effects of visual art therapy on positive symptoms (SMD = 0.293, 95% CI = 0.088 to 0.498, *p* = 0.005), negative symptoms (SMD = 0.742, 95% CI = 0.549 to 0.934, *p* < 0.001), depression (SMD = 0.505, 95% CI = 0.234 to 0.776, *p* < 0.001), and anxiety (SMD = 0.937, 95% CI = 0.286 to 1.587, *p* = 0.005) in the studies published after 2014. The subgroup analysis based on continent revealed that visual art therapy resulted in significant improvements in positive symptoms (SMD = 0.421, 95% CI = 0.218 to 0.624, *p* < 0.001), negative symptoms (SMD = 0.749, 95% CI = 0.563 to 0.936, *p* < 0.001), depression (SMD = 0.646, 95% CI = 0.426 to 0.866, *p* < 0.001), and anxiety (SMD = 0.909, 95% CI = 0.386 to 1.433, *p* = 0.001) in the Asia region. However, there were only marginally significant improvements in positive symptoms (SMD = 0.340, 95% CI = −0.006 to 0.686, *p* = 0.054) and negative symptoms (SMD = 0.399, 95% CI = −0.059 to 0.857, *p* = 0.088) in studies conducted in European countries. Additionally, only one study conducted in Europe investigated emotional aspects, finding no significant improvement in depression (SMD = 0.168, 95% CI = −0.374 to 0.709, *p* = 0.554). The results of the subgroup analysis are presented in Table 1.

For positive symptoms, negative symptoms, depression, and anxiety, meta-regressions were performed for average age, female percentage, duration of illness, and weekly duration of intervention. The findings indicated that a greater amount of intervention (in minutes per week) was associated with a greater improvement in positive symptoms (*β* = 0.001, *p* = 0.004) and was marginally correlated with a greater improvement in negative symptoms (*β* = 0.001, *p* = 0.060). Additionally, a higher proportion of females was associated with better therapeutic effects on negative symptoms (*β* = 1.069, *p* = 0.035) and anxiety (*β* = 5.318, *p* = 0.001). However, none of the other interventions or sample characteristics were associated with treatment effects (all *p* > 0.1). The results of the meta-regression are presented in Table S3.

### 3.5. Certainty of Evidence

The certainty of evidence based on the GRADE system is presented in Table S4. According to the GRADE system, the certainty of evidence for positive symptoms and anxiety disorders were rated as ‘very low’ due to the risk of bias, high heterogeneity, and publication bias. Conversely, the certainty of evidence for negative symptoms and depression were rated as ‘low’ due to the risk of bias and high heterogeneity.

## 4. Discussion

The results of our investigation indicate that visual art therapy results in significant improvements in positive symptoms, negative symptoms, depression, and anxiety among patients with schizophrenia. The subgroup analysis revealed significant therapeutic effects presented by painting, handcrafts, and the combination of painting with calligraphy on psychiatric symptoms and emotions. Additionally, the meta-regression suggested that a longer duration of therapy, as measured in minutes per week, was associated with better therapeutic effects on positive symptoms. Meanwhile, a higher proportion of female participants was associated with better improvement in negative symptoms and anxiety. Our results support the application of visual art therapy in schizophrenia patients to enhance recovery; however, the certainty of the evidence suggests that the results remain inconclusive. More rigorous clinical trials employing appropriate designs are warranted to address the factors of selection bias, performance bias and reporting bias.

This study revealed a small-to-moderate effect (SMD = 0.407) of visual art therapy on positive symptoms. A previous meta-analysis enrolled six RCTs to investigate the effects of art therapy on positive symptoms in patients with schizophrenia, reporting a small, non-significant effect (SMD = −0.32, 95% CI: −0.72 to 0.07) [27]. A separate meta-analysis including six RCTs also found a small, non-significant effect on positive symptoms (*g* = −0.10, 95% CI: −0.35 to 0.15) [26]. Our study included a greater sample size and provides updated evidence favoring visual art therapy. Although the literature included in our analysis may be at risk of publication bias, upon adjustment, the effect sizes remained statistically significant, albeit diminished to a lower magnitude of effect. This provides evidence supporting the effectiveness of including activities such as painting, handcrafts, or combined painting and calligraphy in the psychiatric setting as an adjunctive approach with medication to help patients with schizophrenia cope with their positive symptoms.

The finding that a longer duration of visual art therapy is associated with better treatment outcomes is consistent with previous research suggesting that longer durations of art therapy may yield superior therapeutic effects [17]. Prior studies have also proposed that participants require extended periods to master artistic techniques and to establish appropriate therapeutic relationships [17]. Thus, longer weekly treatment courses may be a contributing factor to the effectiveness of visual art therapy.

Our investigation further found that visual art therapy had a moderate effect on negative symptoms (SMD = 0.697) in patients with schizophrenia. A previous meta-analysis, which included six RCTs of art therapy, identified a small effect size for negative symptoms (SMD = −0.39, 95% CI: −0.68 to −0.09) [27]. Similarly, another meta-analysis that included nine RCTs of art therapy reported a small effect size for negative symptoms (*g* = −0.42, 95% CI: −0.70 to −0.14). Here, we included a greater number of clinical trials on visual art therapy, and the results are consistent with previous findings. However, with an increased sample size, we found that visual art therapy had a moderate effect on negative symptoms, while the results were more precise compared to previous studies. It should be noted that a lack of verbal expression and social interaction are common negative symptoms of schizophrenia, while art therapy has long been recognized as a therapeutic modality that can facilitate engagement when verbal expression is difficult [25]. Art therapy can thus assist individuals with schizophrenia to connect and communicate with themselves, peers, therapists, and the external environment more effectively. Through the process of creating art, individuals with schizophrenia can express themselves in a non-verbal manner, thereby facilitating the healing process [17,66].

We found here that visual art therapy has a moderate effect size on depression (SMD = 0.610) and a high effect size on anxiety (SMD = 0.909) in patients with schizophrenia. According to our research, this study is the first integrated analysis of visual art therapy for the symptoms of depression and anxiety in schizophrenia patients, aligning with previous meta-analyses exploring the effects of art therapy on various populations [28]. Of note, a previous review indicated that art therapy may effectively alleviate anxiety in prisoners [67], while a separate study demonstrated that art therapy can effectively reduce symptoms of anxiety and depression in cancer patients [68]. Visual art therapy, such as painting, has long been embraced across diverse social and cultural contexts, providing participants with a means to express their fears and worries through the process of artistic creation, thereby ameliorating depression and anxiety [20]. Similarly, our findings suggest that visual art therapy may serve as an effective adjunctive treatment option for individuals with schizophrenia when combined with antidepressants and mood stabilizers, warranting further investigation.

Further noted in our investigation was that a higher percentage of females were associated with greater improvements in negative symptoms and anxiety. These findings are consistent with previous research examining negative symptoms in schizophrenia patients [26]. In addition, prior studies have supported the efficacy of art therapy in reducing anxiety symptoms in female cancer patients [69]. Furthermore, reports have indicated that the majority of art therapists are female [70], and that females are often the predominant users of art therapy [71]. Therefore, female patients with schizophrenia may be more responsive to visual art therapies, a factor warranting further investigation in future studies.

The results of our subgroup analysis indicated that art therapy in the form of painting is effective for psychiatric symptoms as well as emotional outcomes in individuals with schizophrenia compared to control conditions. This finding corresponds to the notion that painting is the primary modality for art therapy in schizophrenia patients [20]. Similarly, a previous study suggested that combining medication with painting therapy significantly improves patient compliance and self-awareness in individuals with schizophrenia [20].

In terms of specific modalities, we herein reveal that handcrafts and combined painting and calligraphy presented benefits for alleviating the psychiatric symptoms, depression, and anxiety associated with schizophrenia. This provides preliminary evidence supporting the efficacy of different types of visual art therapy in the treatment of schizophrenia. Similarly, previous meta-analyses have investigated the use of calligraphy activities in participants with various diagnoses, noting significant effects [28]. In our study, we further differentiated between calligraphy activities combined with painting and calligraphy activities used alone for treating schizophrenia. The results suggest that using calligraphy activities alone does not have statistically significant effects, which may be attributed to the limited sample size (only two studies applied calligraphy alone) and requires further investigation.

In this study, subgroup analyses were conducted based on publication years. The results indicated that regardless of whether the studies were published before or after 2014, visual art therapy showed improvements in positive symptoms, negative symptoms, depression, and anxiety in individuals with schizophrenia. The number of relevant studies increased after 2014; this is consistent with findings of previous studies discussing the quantity of research publications on art therapy [20], and highlights the potential application of visual art therapy. This study also noted that the effectiveness of visual art therapy was better in Asian (particularly East Asian) compared to European populations. Previous research has also reported better functional remission in schizophrenia in the East Asian region, possibly related to cultural factors and family support [72,73]. In terms of visual art therapy, art therapists in Chinese culture may integrate traditional Chinese Daoist philosophy [74], thus incorporating elements of mindfulness meditation into the process of painting and crafts creation to promote recovery. However, most of the literature in this study was published in East Asia, while the European literature showed marginal significance (*p* values: 0.054 to 0.088). Therefore, future research on the application of visual art therapy for schizophrenia in different cultural regions is necessary.

## 5. Conclusions

Taken together, these studies indicate that visual art therapy can improve positive symptoms, negative symptoms, depression, and anxiety in patients with schizophrenia. It can serve as an effective adjunctive therapy alongside appropriate pharmacological treatments. Additionally, a longer weekly treatment duration appears to be more beneficial for improving positive symptoms. Moreover, visual art therapy may be particularly helpful for alleviating negative symptoms and anxiety in female schizophrenia patients. Various forms of visual art therapy, including painting, handcrafts, and painting combined with calligraphy, demonstrate significant efficacy. There are several limitations to this systematic review and analysis. First, most of the included literature lacked clinical trial registration. Additionally, there were numerous risks of bias in the studies, leading to a decreased certainty of evidence, and suggesting that the results should be interpreted with caution. Another limitation is that this study did not investigate the benefits of visual art therapy on aspects such as functional capacity and quality of life, which are crucial for the recovery of individuals with schizophrenia. These aspects warrant further exploration in future research. Finally, future studies employing more rigorous designs, such as blinded outcome assessments, more detailed allocation processes, and trial registration, are recommended. Further investigations into visual art therapy in different cultural regions are also warranted. In summary, we need to interpret these study results cautiously, as further rigorous clinical trials are required to validate our findings.

## Figures and Tables

**Table 1:** Outcomes and subgroup analysis.

			Effect Size			Heterogeneity		
	Studies	Total n	SMD	95% CI	*p* Value	*Q* Value	*p* Value	*I*^2^ %
**Visual art**								
Positive symptoms	22	2033	0.407	0.233–0.581	<0.001	77.689	<0.001	72.969
Negative symptoms	26	2539	0.697	0.514–0.880	<0.001	123.222	<0.001	79.711
Depression	11	973	0.610	0.398–0.821	<0.001	25.848	0.004	61.313
Anxiety	6	527	0.909	0.386–1.433	0.001	40.240	<0.001	87.575
**Painting**								
Positive symptoms	14	1373	0.333	0.153–0.513	<0.001	34.829	0.001	62.675
Negative symptoms	17	1832	0.704	0.510–0.897	<0.001	62.628	<0.001	74.452
Depression	6	545	0.681	0.425–0.938	<0.001	10.344	0.066	51.662
Anxiety	3	223	1.317	0.428–2.207	0.004	17.115	<0.001	88.314
Handcrafts *								
Positive symptoms	3	267	0.492	0.239–0.744	<0.001	2.317	0.344	6.397
Negative symptoms	4	317	1.076	0.374–1.777	0.003	24.873	<0.001	87.939
Depression	1	64	0.681	0.177–1.185	0.008	0.000	>0.999	0.000
**Calligraphy**								
Positive symptoms	1	137	−0.265	−0.601–0.072	0.123	0.000	>0.999	0.000
Negative symptoms	1	137	0.023	−0.312–0.358	0.895	0.000	>0.999	0.000
Depression	2	235	0.313	−0.386–1.011	0.380	7.025	0.008	85.765
Anxiety	2	235	0.412	−0.413–1.236	0.328	9.612	0.002	89.597
**Combined painting and calligraphy**								
Positive symptoms	4	253	0.824	0.225–1.422	0.007	15.817	0.001	81.033
Negative symptoms	4	253	0.491	−0.059–1.042	0.080	14.116	0.003	78.748
Depression	2	129	0.712	0.356–1.069	<0.001	0.853	0.356	0.000
Anxiety	1	69	0.840	0.347–1.332	0.001	0.000	>0.999	0.000
**Publishing year before 2014**								
Positive symptoms	9	851	0.578	0.272–0.883	<0.001	36.465	<0.001	78.061
Negative symptoms	9	845	0.615	0.231–0.999	0.002	56.758	<0.001	85.905
Depression	4	313	0.801	0.568–1.035	<0.001	3.072	0.381	2.351
Anxiety	1	98	0.841	0.428–1.255	<0.001	0.000	>0.999	0.000
**Publishing year after 2014**								
Positive symptoms	13	1179	0.293	0.088–0.498	0.005	36.592	<0.001	67.206
Negative symptoms	17	1694	0.742	0.549–0.934	<0.001	57.413	<0.001	72.132
Depression	7	660	0.505	0.234–0.776	<0.001	17.088	0.009	64.888
Anxiety	5	429	0.937	0.286–1.587	0.005	39.528	<0.001	89.881
**Continent of publication—Asia**								
Positive symptoms	18	1598	0.421	0.218–0.624	<0.001	68.350	<0.001	75.128
Negative symptoms	22	2107	0.749	0.563–0.936	<0.001	88.734	<0.001	76.334
Depression	10	920	0.646	0.426–0.866	<0.001	23.620	0.005	61.896
Anxiety	6	527	0.909	0.386–1.433	0.001	40.240	<0.001	87.575
**Continent of publication—Europe ^$^**								
Positive symptoms	4	432	0.340	−0.006–0.686	0.054	7.943	0.047	62.233
Negative symptoms	4	432	0.399	−0.059–0.857	0.088	13.719	0.003	78.132
Depression	1	53	0.168	−0.374–0.709	0.544	0.000	>0.999	0.000

SMD: standardized mean difference. * There were insufficient data regarding the effects of handcrafts on anxiety to perform a subgroup analysis. ^$^ There were insufficient data published in Europe regarding the effects on anxiety to perform a subgroup analysis.

## Data Availability

The raw data supporting the conclusions of this article will be made available by the authors on request.

## Supplementary Materials

The following supporting information can be downloaded at: https://www.mdpi.com/article/10.3390/healthcare12111156/s1, Figure S1: Funnel plot of positive symptoms. Figure S2: Funnel plot of negative symptoms. Figure S3: Funnel plot of depression. Figure S4: Funnel plot of anxiety. Figure S5: Subgroup analysis of painting for positive symptoms. Figure S6: Subgroup analysis of painting for negative symptoms; subgroup analysis of painting for depression. Figure S8: Subgroup analysis of painting for anxiety. Figure S9: Subgroup analysis of crafts for positive symptoms. Figure S10: Subgroup analysis of crafts for negative symptoms. Figure S11: Subgroup analysis of crafts for depression. Figure S12: Subgroup analysis of calligraphy for positive symptoms. Figure S13: Subgroup analysis of calligraphy for negative symptoms. Figure S14: Subgroup analysis of calligraphy for depression. Figure S15: Sub-group analysis of calligraphy for anxiety. Figure S16: Subgroup analysis of painting and calligraphy for positive symptoms. Figure S17: Subgroup analysis of painting and calligraphy for negative symptoms. Figure S18: Subgroup analysis of painting and calligraphy for depression. Figure S19: Subgroup analysis of painting and calligraphy for anxiety. Table S1: Search texts and strategies. Table S2: Details of included studies. Table S3: Meta-regression for examining moderator relationships. Table S4: GRADE summary table.
