# Supplementary material for: Effects of Visual Art Therapy on Positive Symptoms, Negative Symptoms, and Emotions in Individuals with Schizophrenia: A Systematic Review and Meta-Analysis
Source: Healthcare (Basel). 2024 Jun 6;12(11):1156. doi: 10.3390/healthcare12111156 (PMC11171575; doi:10.3390/healthcare12111156)
Supplement: Supplementary file 1 [file healthcare-12-01156-s001.zip › healthcare-2970463-supplementary.pdf]

Supplemental data

Effects of Art Therapy on Positive Symptoms, Negative Symptoms and Emotions in Schizophrenia: A Systematic Review and Meta-analysis

Table S1. Search texts and strategies

Database	Search terms	Filters
PubMed	(Schizophrenia OR "Severe mental illness" [Title/Abstract]) AND ("art therap*" OR "Art Therapy" [Mesh] OR draw* OR sketching OR craft OR calligraphic OR paint* OR Colouring OR Sandpainting OR Collage making OR artistic pottery OR sculpting)	Title/Abstract; Randomized controlled trial
Embase	('schizophrenia'/exp OR 'severe mental illness'/exp) AND ('art therapy'/exp OR draw* OR sketching OR craft OR calligraphic OR paint* OR Colouring OR Sandpainting OR Collage making OR artistic pottery OR sculpting)	Title/Abstract; Randomized controlled trial
Cochrane Library	(Schizophrenia OR (MeSH descriptor: [Schizophrenia] explode all trees) OR severe mental illness) AND ((MeSH descriptor: [Art Therapy] explode all trees) OR draw* OR sketching OR craft OR calligraphic OR paint* OR Colouring OR Sandpainting OR Collage making OR artistic pottery OR sculpting)	Title/Abstract; Trials
CEPS	((([ALL]=(思覺失調) OR [ALL]=(精神分裂)) OR [ALL]=(精障)) AND ([ALL3]=(繪畫) OR [ALL3]=(藝術) OR [ALL3]=(書法) OR [ALL3]=(手工藝) OR [ALL3]=(沙畫) OR [ALL3]=(雕塑)))	N/A
CNKI	(精神分裂 OR 精障) AND (繪畫OR藝術OR書法OR手工藝OR沙畫 OR 雕塑)	Title/Keywords
Wanfang database	(精神分裂 OR 精障) AND (繪畫OR藝術OR書法OR手工藝OR沙畫 OR 雕塑)	N/A
Yiigle	(精神分裂 OR 精障) AND (繪畫OR藝術OR書法OR手工藝OR沙畫 OR 雕塑)	N/A

Table S2. Details of included studies

Study (country)	Sample Size (setting)	Age (mean ± SD)	Experimental condition	Control condition	Supervision/ Form	Outcome Measures *Primary	Duration of Intervention
Huang 2020 [59] (China)	AT: 32 Con: 32 (inpatient)	AT: 30.61 ± 1.08 Con: 30.52 ± 1.04	A combination of painting and calligraphy	Conventional therapy	Nurse/Group	PANSS	90 mins/session, 2 sessions/week, not mentioned how long it last
Chen 2021 [62] (China)	AT: 32 Con: 32 (inpatient)	AT: 37.97 ± 13.01 Con: 43.63 ± 11.61	Crafts	Conventional therapy	Nurse/Group	HAMD, BPRS	60 mins/session, 5 sessions/week, lasted 6 weeks. Total: 1800 mins
Chen 2016 [37] (Taiwan)	AT: 22 Con: 22 (inpatient)	AT: 44.6 ± 11.8 Con: 46.1 ± 12.4	Painting	Conventional therapy	Nurse/Group	SIAS	60 mins/session, 1 session/week, lasted 8 weeks. Total: 480 mins
Meng 2005 [53] (China)	AT: 43 Con: 43 (inpatient)	AT: 35.14 ± 10.12 Con: 37.58 ± 12.77	Painting	Conventional therapy	Researcher/Grou p	PANSS	120 mins/session, 2 sessions/week, lasted 15 weeks. Total: 3600 mins
Li 2020 [58] (China)	AT: 39 Con: 39 (inpatient)	AT: 45.95 ± 5.28 Con: 46.12 ± 5.27	Painting	Conventional therapy	Art therapist/Group	SAS, SDS, PANSS	60 mins/session, 2 sessions/week, lasted 6 weeks. Total: 720 mins
Yuan 2023 [42] (China)	AT: 60 Con: 60 (inpatient)	AT: 39.46 ± 5.75 Con: 38.92 ± 6.03	Painting	Conventional therapy	Nurse/Group	PANSS	60-90 mins/session, 2 sessions/week, lasted 12 weeks. Total: 1800 mins
Dong 2013 [60] (China)	AT: 35 Con: 34 (inpatient)	AT: 32 ± 7 Con: 31 ± 9	A combination of painting and calligraphy	Conventional therapy	Therapist/Group	PANSS, SCL-90	Not mention how long it takes for one session, 2 sessions/week, lasted 12 weeks.
You 2023 [43] (China)	AT: 47 Con: 47 (inpatient)	AT: 46.63 ± 6.31 Con: 48.15 ± 8.31	Painting	Routine nursing intervention (Aripiprazole)	Nurse/Group	PANSS	90 mins/session, 2 sessions/week, lasted 15 weeks. Total: 2700 mins
Crawford 2012 [35] (UK)	AT: 140 Con: 137 (inpatient)	AT: 41 ± 11 Con: 40 ± 12	Painting	Conventional therapy	Art therapist/Group	PANSS	90 mins/session, 1 sessions/week, lasted 51.43 weeks. Total: 4628.7 mins
Montag 2014 [39] (Germany)	AT: 29 Con: 24 (inpatient)	AT: 37.4 ± 11.2 Con: 38.8 ± 10.4	Painting	TAU	Art therapist/Group	SANS, SAPS, CDSS	90 mins/session, 2 sessions/week, lasted 6 weeks. Total: 1080 mins
Li 2022 [57] (China)	AT: 30 Con: 30 (inpatient)	AT: 45.1 ± 5.25 Con: 44.65 ± 6.11	Painting	Conventional therapy	Psychiatrist& nurse/Group	PANSS, BPRS, NOSIE	90 mins/session, 2 sessions/week, lasted 15 weeks. Total: 2700 mins
Qiu 2017 [52] (China)	AT: 54 Con: 51 (prison)	ALL:37.8 ± 14.5	Painting	Conventional therapy	Art therapist/Group	STAXI, BDI, PANSS	120 mins/session, 1 sessions/week, lasted 48 weeks. Total: 5760 mins
Xiong 2016 [45] (China)	AT: 30 Con: 30 (inpatient)	AT: 39.6 ± 7.1 Con: 38.2 ± 10.5	Painting	Conventional therapy	Art therapist/Group	BPRS, SANS	60 mins/session, 1 sessions/week, lasted 12.86 weeks. Total: 771.6 mins
Xu 2022 [44] (China)	AT: 133 Con: 132 (inpatient)	AT: 43.72 ± 5.2 Con: 43.67 ± 5.12	Painting	Conventional therapy	Not mentioned /Group	PANSS	90 mins/session, 2 sessions/week, lasted 15 weeks. Total: 2700 mins

Chen 2020 [64] (China)	AT: 47 Con: 48 (inpatient)	AT: 30.65 ± 7.65 Con: 31.31 ± 8.88	Painting	Antipsychotic drugs and work entertainment treatment	Not mentioned /Group	SANS	Not mentioned how long it takes and how many sessions a week, lasted 12.86 weeks.
Liang 2016 [54] (China)	AT: 60 Con: 60 (inpatient)	AT: 41.43 ± 12.18 Con: 40.87 ± 11.86	Painting	Conventional therapy	Art therapist/Group	BPRS	120 mins/session, 4 sessions/week, lasted 8 weeks. Total: 3840 mins
Tong 2019 [51] (China)	AT: 53 Con: 51 (inpatient)	AT: 43.68 ± 7.25 Con: 46.06 ± 8.26	Painting	Conventional therapy	Nurse/Group	PANSS	90 mins/session, 2 sessions/week, lasted 15 weeks. Total: 2700 mins
Wang 2021 [48] (China)	AT: 57 Con: 57 (inpatient)	AT: 43.27 ± 4.84 Con: 42.85 ± 5.2	Painting	Conventional therapy	Nurse/Group	PANSS	90 mins/session, 2 sessions/week, lasted 15 weeks. Total: 2700 mins
Li 2010 [55] (China)	AT: 30 Con: 30 (inpatient)	AT: 32.6 ± 11.3 Con: 32.1 ± 11.2	A combination of painting and calligraphy	Not mentioned	Nurse/Group	SANS, BPRS, NOSIE	60-120 mins/session, 1 sessions/week, lasted 8 weeks. Total: 720 mins
Wang 2020 [47] (China)	AT: 80 Con: 80 (inpatient)	AT: 40.5±6.3 Con: 39.9±6.2	Painting	Antipsychotic drugs and work entertainment treatment	Not mentioned /Group	PANSS, NOSIE	120 mins/session, 3 sessions/week, lasted 12 weeks. Total: 4320 mins
Huang 2022 [38] (Taiwan)	AT: 76 Con: 74 (inpatient)	AT: 46.68 ± 9.35 Con: 46.59 ± 10.45	Calligraphy	TAU	Occupational therapist/Group	DASS-21, PANSS	70 mins/session, 1 sessions/week, lasted 24 weeks. Total: 1680 mins
Richardson 2007 [36] (UK)	AT: 43 Con: 47 (mental health service users)	AT: 39.6 ± 10.5 Con: 42.6 ± 11.5	Painting	Standard Psychiatric Care (SPC)	Not mentioned /Group	SANS, BPRS	90 mins/session, 1 sessions/week, lasted 12 weeks. Total: 1080 mins
Utas-Akhan [40] 2023 (Turkey)	AT: 32 Con: 34 (Community mental health center)	Not mentioned	Painting	Conventional therapy	Art therapist/Group	PANSS	60 mins/session, 2 sessions/week, lasted 10 weeks. Total: 1200 mins
Cao 2006[65] (China)	AT: 48 Con: 50 (inpatient)	AT: 25 ± 8.6 Con: 30 ± 12.3	Calligraphy	Conventional therapy	Not mentioned /Group	SCL-90	60 mins/session, 5 sessions/week, lasted 8 weeks. Total: 2400 mins
Chen 2005 [63] (China)	AT: 30 Con: 30 (inpatient)	AT: 28.21 ± 7.67 Con: 30.61 ± 9.31	Crafts	Conventional therapy	Nurse/Group	SANS	Not mention how long it takes for one session, 1 sessions/week, lasted 8 weeks.
Cheng 2021 [61] (China)	AT: 30 Con: 30 (inpatient)	AT: 37.59 ± 2.48 Con: 37.82 ± 2.34	Crafts	No intervention	Nurse/Group	PANSS	Not mentioned how long it takes and how many sessions a week, lasted 8 weeks.
Wang 2005 [50] (China)	AT: 57 Con: 60 (inpatient)	AT: 28.31 ± 8.26 Con: 29.33 ± 7.83	Crafts	Conventional therapy	Nurse/Group	CRDPSS, SANS	180 mins/session, 3 sessions/week, lasted 12 weeks. Total: 6480 mins
Wang 2012 [49] (China)	AT: 43 Con: 43 (inpatient)	Not mentioned	Crafts	Conventional therapy	Nurse/Group	BPRS	90-120 mins/session, 6 sessions/week, lasted 20 weeks. Total: 12600 mins

Liang 2016 [54] (China)	AT: 40 Con: 40 (inpatient)	AT: 32.72 ± 10.02 Con: 33.5 ± 9.18	Crafts	Conventional therapy	Nurse/Group	SANS	90 mins/session, 10 sessions/week, lasted 8 weeks. Total: 7200 mins
Zhou 2010 [41] (China)	AT: 30 Con: 30 (inpatient)	AT: 40.38 ± 11.2 Con: 40.38 ± 11.2	A combination of painting and calligraphy	Conventional therapy	Art therapist/Group	BPRS, SANS	120 mins/session, 10 sessions/week, lasted 12.86 weeks. Total: 15432 mins
Li 2017 [56] (China)	AT: 38 Con: 40 (community)	AT: 47.26 ± 6.11 Con: 49.58 ± 9.9	Painting	Conventional therapy	Art therapist/Group	PANSS	45 mins/session, 2 sessions/week, lasted 25.71 weeks. Total: 2313.9 mins

AT, art therapy; Con, control; PANSS, Positive and Negative Symptoms Scale; HAMD, Hamilton Depression Scale; BPRS, Brief Psychiatric Rating Scale; SIAS, Social Interaction Anxiety Scale; SAS, Self-Rating Anxiety Scale; SDS, Self-rating Depression Scale; SCL-90, self－rating symptom scale; SANS, Scale for the assessment of negative symptoms; SAPS, Scale for the assessment of positive symptoms; CDSS, Calgary depression scale for schizophrenia; NOSIE, psychiatric symptom rating scale-nurse inpatient observation scale; STAXI, state–trait anger expression inventory; BDI, Beck depression inventory; DASS-21, Depression, Anxiety, and Stress Scale; CRDPSS, Clinician-Rated Dimensions of Psychosis Symptom Severity; NOSIE, nurse observation scale for inpatient evaluation.

Table S3. Meta-regression for examining moderator relationships.

Outcome	Moderator	Studies	β	*p* value
Positive symptoms	Mean age	20	-0.023	.153
	Female percentage	22	0.017	.971
	Illness years	19	-0.022	.204
	**Weekly minutes**	**21**	**0.001**	**.004***
Negative symptoms	Mean age	25	0.016	.343
	**Female percentage**	**26**	**1.069**	**.035***
	Illness years	22	-0.001	.950
	Weekly minutes	22	0.0001	.060
Depression	Mean age	11	-0.019	.271
	Female percentage	11	0.207	.729
	Illness years	10	-0.006	.715
	Weekly minutes	10	0.0005	.701
Anxiety	Mean age	6	0.013	.738
	**Female percentage**	**6**	**5.3181**	**.001**
	Illness years	5	-0.017	.730
	Weekly minutes	5	-0.001	.808

Table S4. GRADE summary table

Visual art therapy compared to control for schizophrenia					
Outcomes	№ of participants (studies) Follow-up	Certainty of the evidence (GRADE)	Relative effect (95% CI)	Anticipated absolute effects	
				Risk with control	Risk difference with Visual art therapy
Changes in positive symptom assessed with: PANSS, SAPS,BPRS,CRDPSS	(22 RCTs)	⊕◯◯◯ Very low	-	-	SMD **0.407 SD higher** (0.233 higher to 0.697 higher)
Change in negative symptom assessed with: PANSS, SANS,	(26 RCTs)	⊕⊕◯◯ Low	-	-	SMD **0.697 SD higher** (0.514 higher to 0.88 higher)
Change in depression assessed with: HAMD, SDS, BDI, DASS-21, SCL-90, PANSS, NOSIE, CDSS	(11 RCTs)	⊕⊕◯◯ Low	-	-	SMD **0.61 SD higher** (0.398 higher to 0.821 higher)
Change in anxiety assessed with: SIAS, SAS, SCL-90, STAXI, DASS-21	(6 RCTs)	⊕◯◯◯ Very low	-	-	SMD **0.909 SD higher** (0.386 higher to 1.433 higher)

*__The risk in the intervention group__ (and its 95% confidence interval) is based on the assumed risk in the comparison group and the **relative effect** of the intervention (and its 95% CI).

CI: confidence interval; **SMD:** standardized mean difference

GRADE Working Group grades of evidence

High certainty: we are very confident that the true effect lies close to that of the estimate of the effect.
Moderate certainty: we are moderately confident in the effect estimate: the true effect is likely to be close to the estimate of the effect, but there is a possibility that it is substantially different.
Low certainty: our confidence in the effect estimate is limited: the true effect may be substantially different from the estimate of the effect.
Very low certainty: we have very little confidence in the effect estimate: the true effect is likely to be substantially different from the estimate of effect.

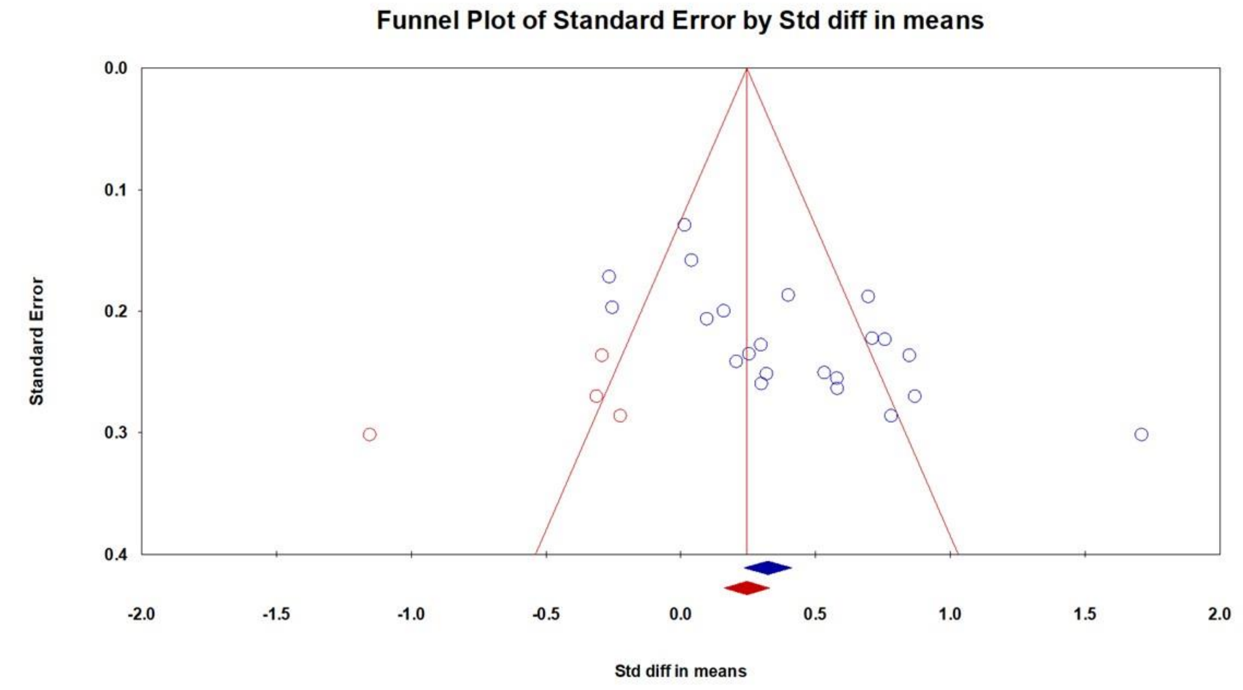

Figure S1. Funnel plot of Positive symptoms

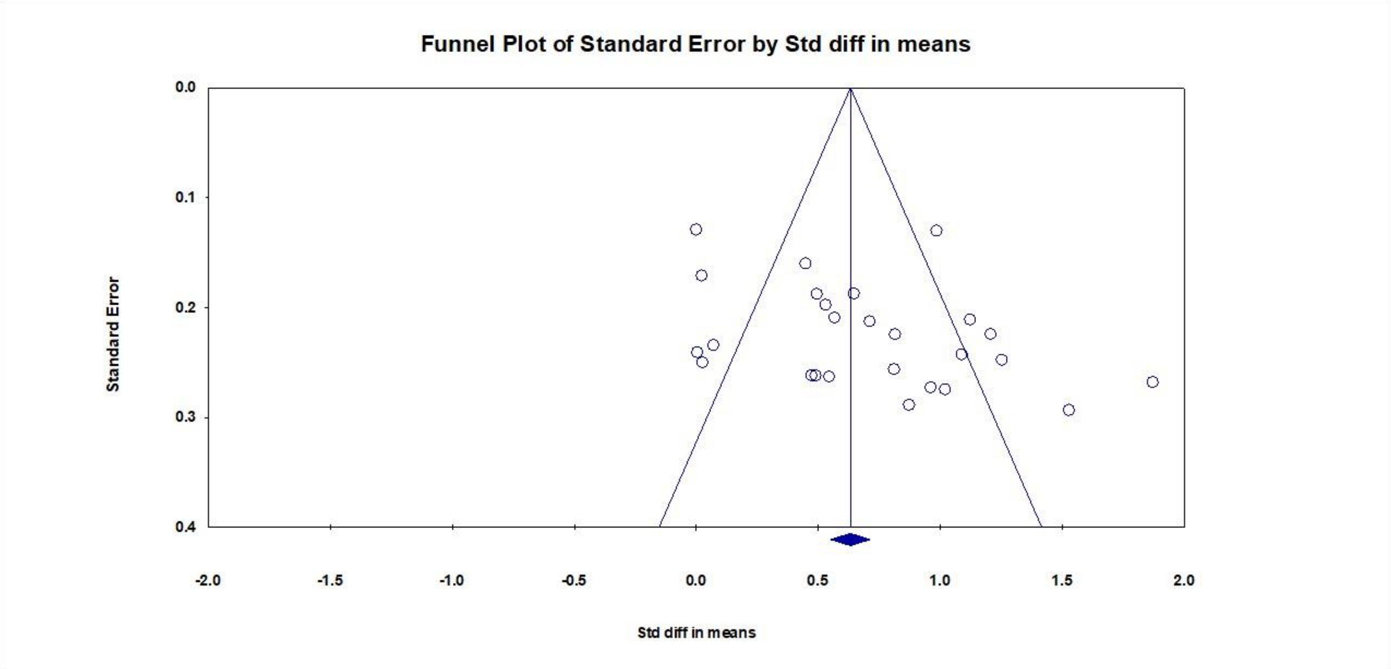

Figure S2. Funnel plot of Negative symptoms

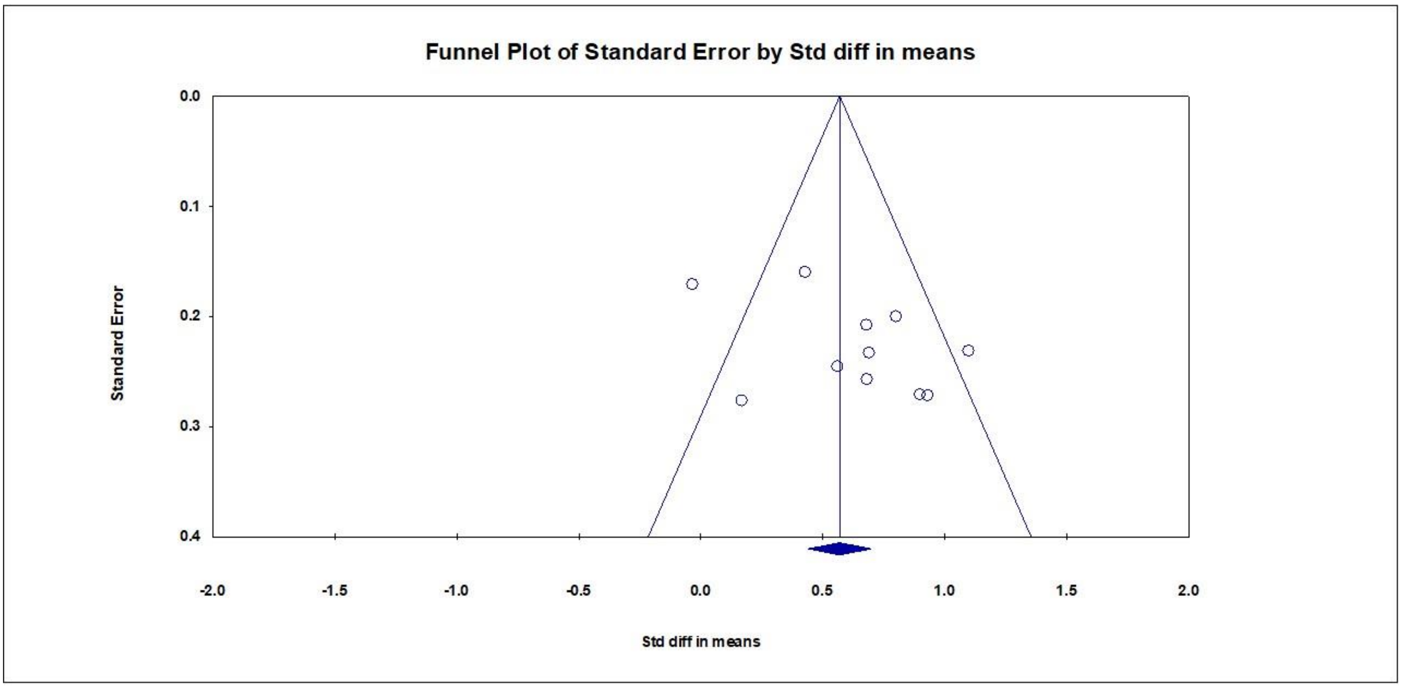

Figure S3. Funnel plot of Depression.

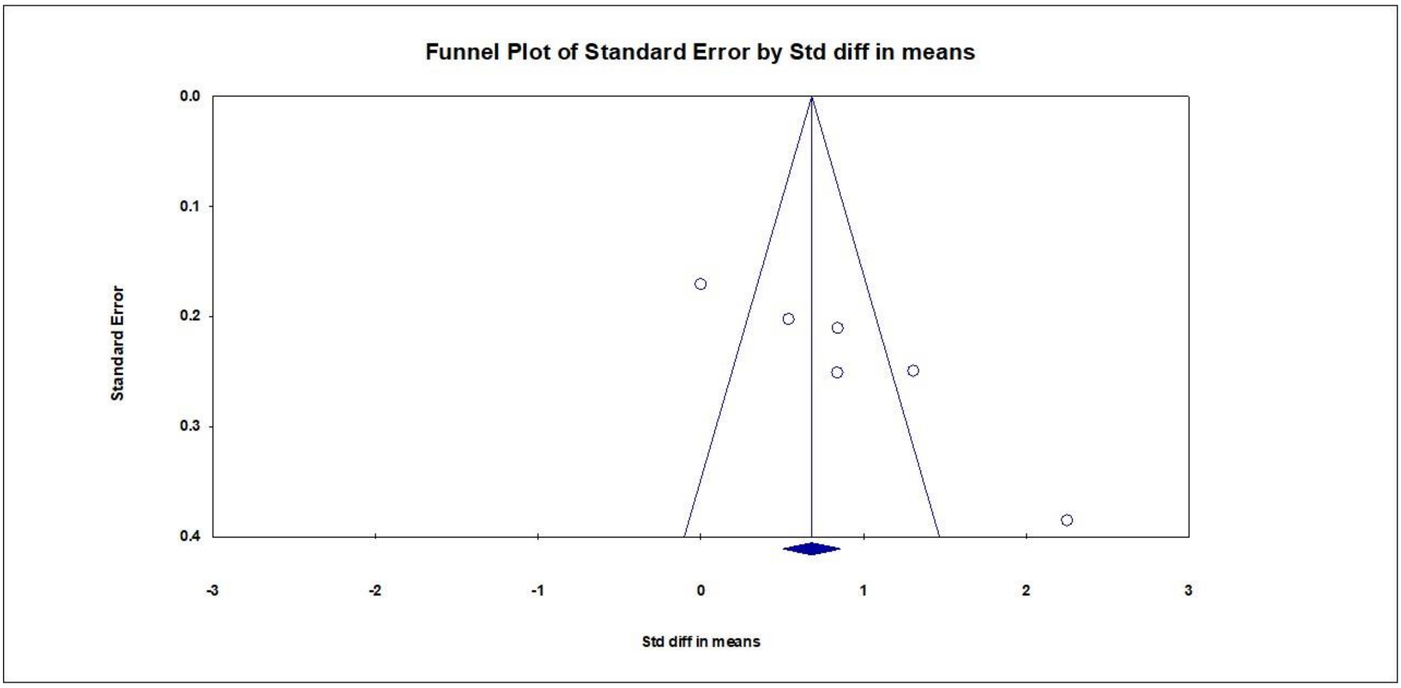

Figure S4. Funnel plot of Anxiety.

Study name	Std diff in means	Lower limit	Upper limit	p-Value
Crawford et al. (2012)	0.015	-0.238	0.268	0.907
Li et al. (2017)	0.298	-0.148	0.744	0.191
Li et al. (2020)	0.848	0.385	1.312	0.000
Li et al. (2022)	0.300	-0.209	0.808	0.249
Meng et al. (2005)	0.710	0.274	1.146	0.001
Montag et al. (2014)	0.781	0.220	1.342	0.006
Qiu et al. (2017)	0.160	-0.232	0.552	0.423
Richardson et al. (2007)	0.253	-0.208	0.715	0.281
Tong et al. (2019)	-0.253	-0.639	0.133	0.198
Utas-Akhan et al. (2023)	0.533	0.042	1.024	0.033
Wang et al. (2020)	0.040	-0.270	0.350	0.799
Xiong et al. (2016)	0.581	0.065	1.098	0.027
You et al. (2023)	0.097	-0.307	0.502	0.637
Yuan et al. (2023)	0.696	0.328	1.065	0.000
Pooled	0.333	0.153	0.513	0.000

Statistics for each study

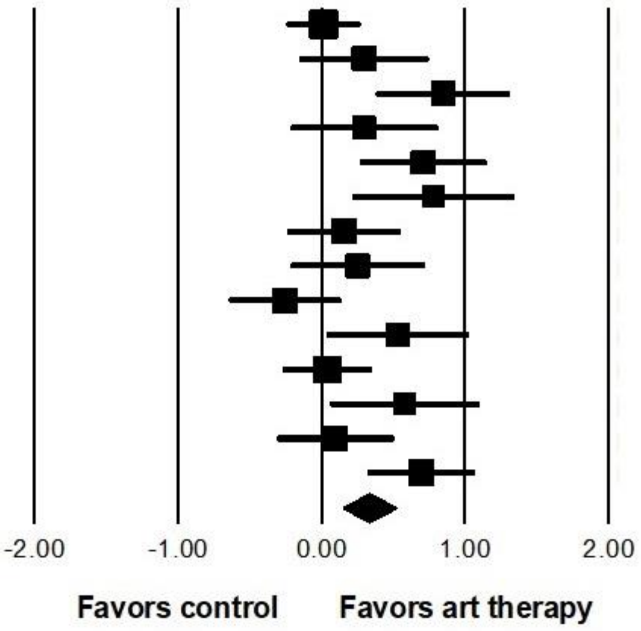

Figure S5. Subgroup analysis of painting for positive symptoms

Study name	Std diff in means	Lower limit	Upper limit	p-Value
Chen et al. (2020)	0.566	0.156	0.977	0.007
Crawford et al. (2012)	0.000	-0.253	0.253	1.000
Li et al. (2017)	1.252	0.767	1.738	0.000
Li et al. (2020)	1.087	0.612	1.563	0.000
Li et al. (2022)	0.489	-0.025	1.003	0.062
Meng et al. (2005)	0.814	0.375	1.254	0.000
Montag et al. (2014)	0.872	0.306	1.438	0.003
Qiu et al. (2017)	0.530	0.143	0.917	0.007
Richardson et al. (2007)	0.070	-0.389	0.529	0.765
Tong et al. (2019)	1.122	0.708	1.536	0.000
Utas-Akhan et al. (2023)	0.811	0.308	1.313	0.002
Wang et al. (2020)	0.448	0.135	0.762	0.005
Wang et al. (2021)	0.711	0.294	1.128	0.001
Xiong et al. (2016)	0.544	0.029	1.060	0.038
Xu et al. (2022)	0.984	0.729	1.239	0.000
You et al. (2023)	1.206	0.766	1.645	0.000
Yuan et al. (2023)	0.646	0.279	1.013	0.001
Pooled	0.704	0.510	0.897	0.000

Statistics for each study

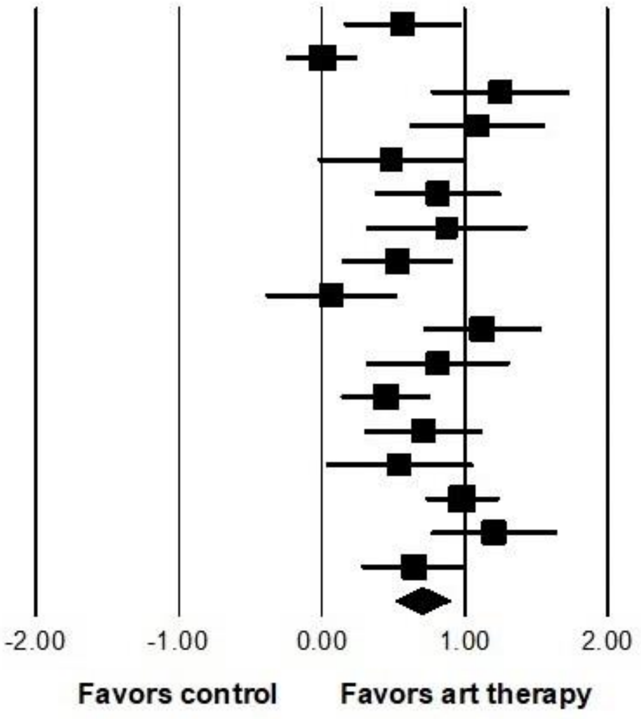

Figure S6. Subgroup analysis of painting for negative symptoms

Study name	Statistics for each study			
	Std diff in means	Lower limit	Upper limit	p-Value
Li et al. (2020)	0.689	0.232	1.146	0.003
Li et al. (2022)	0.929	0.397	1.462	0.001
Meng et al. (2005)	1.097	0.643	1.550	0.000
Montag et al. (2014)	0.168	-0.374	0.709	0.544
Qiu et al. (2017)	0.800	0.408	1.192	0.000
Wang et al. (2020)	0.427	0.114	0.741	0.008
Pooled	0.681	0.425	0.938	0.000

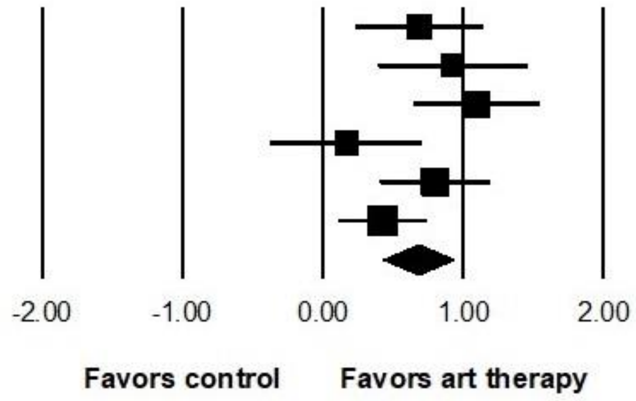

Figure S7. Subgroup analysis of painting for depression.

Study name	Statistics for each study			
	Std diff in means	Lower limit	Upper limit	p-Value
Chen et al. (2016)	2.251	1.496	3.007	0.000
Li et al. (2020)	1.307	0.818	1.796	0.000
Qiu et al. (2017)	0.540	0.143	0.937	0.008
Pooled	1.317	0.428	2.207	0.004

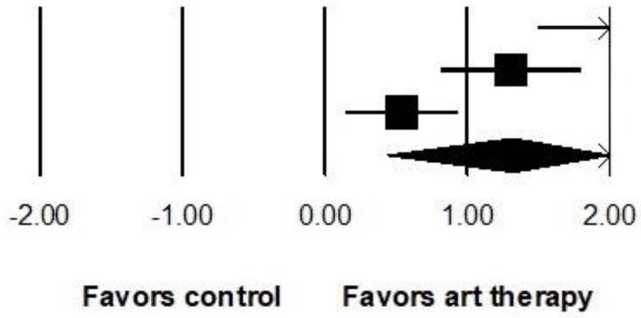

Figure S8. Subgroup analysis of painting for anxiety.

Study name	Statistics for each study			
	Std diff in means	Lower limit	Upper limit	p-Value
Chen et al. (2021)	0.319	-0.174	0.812	0.205
Wang et al. (2005)	0.399	0.033	0.766	0.032
Wang et al. (2012)	0.758	0.320	1.195	0.001
Pooled	0.492	0.239	0.744	0.000

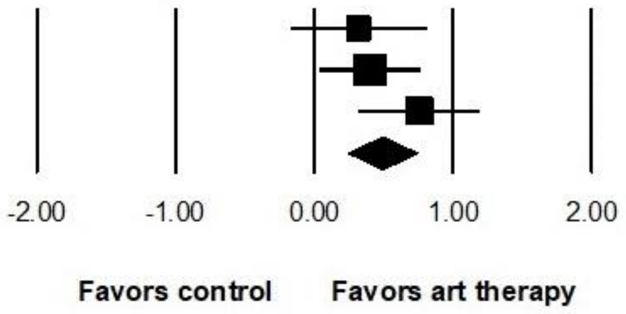

Figure S9. Subgroup analysis of crafts for positive symptoms.

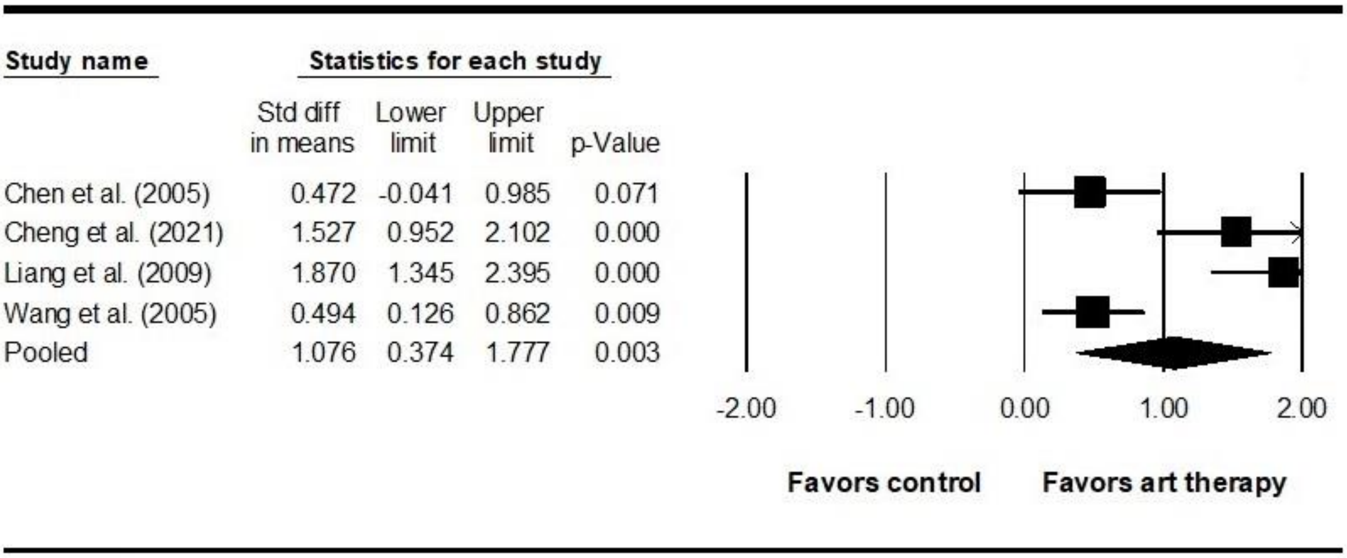

Figure S10. Subgroup analysis of crafts for negative symptoms.

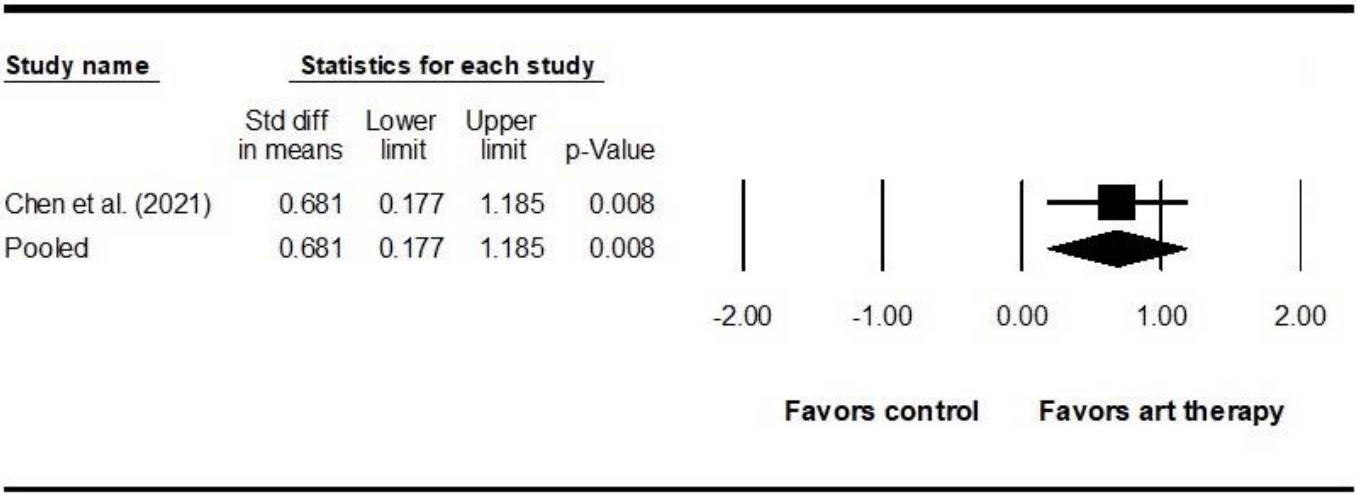

Figure S11. Subgroup analysis of crafts for depression.

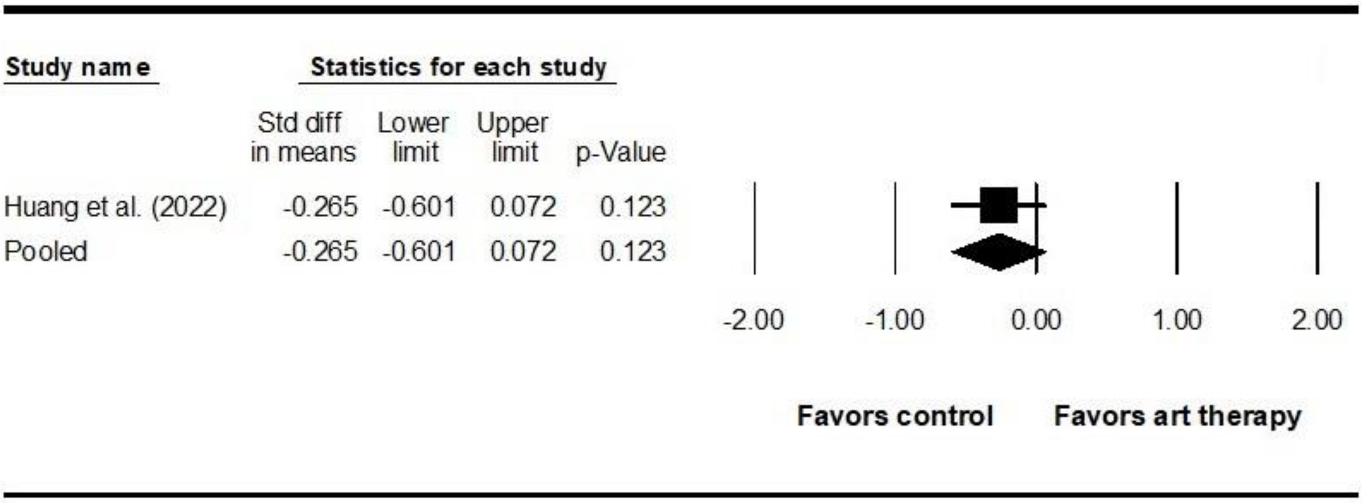

Figure S12. Subgroup analysis of calligraphy for positive symptoms.

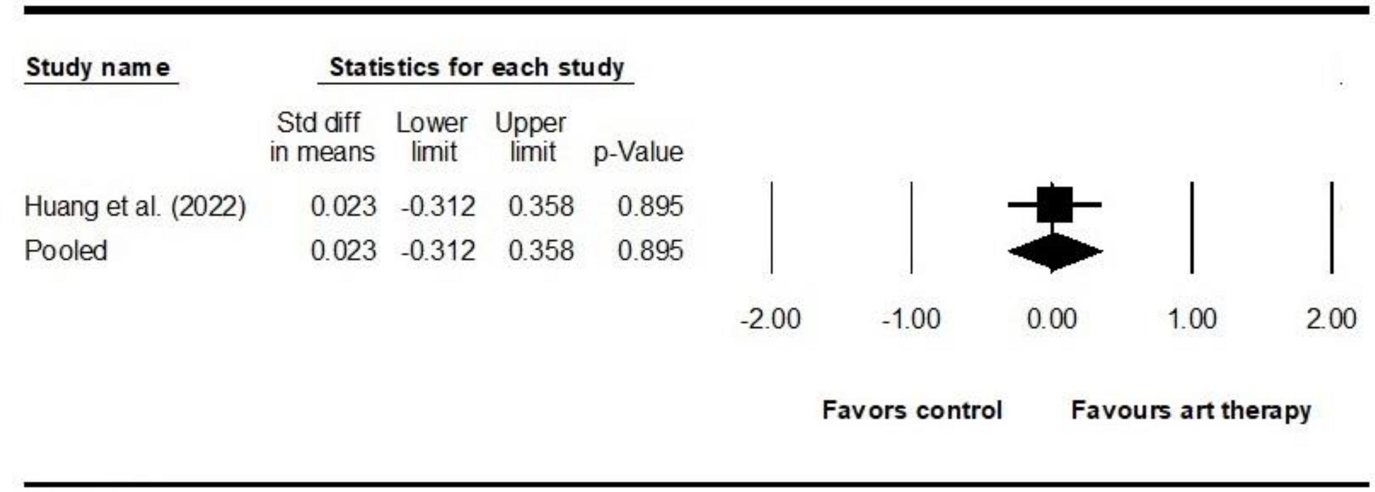

Figure S13. Subgroup analysis of calligraphy for negative symptoms.

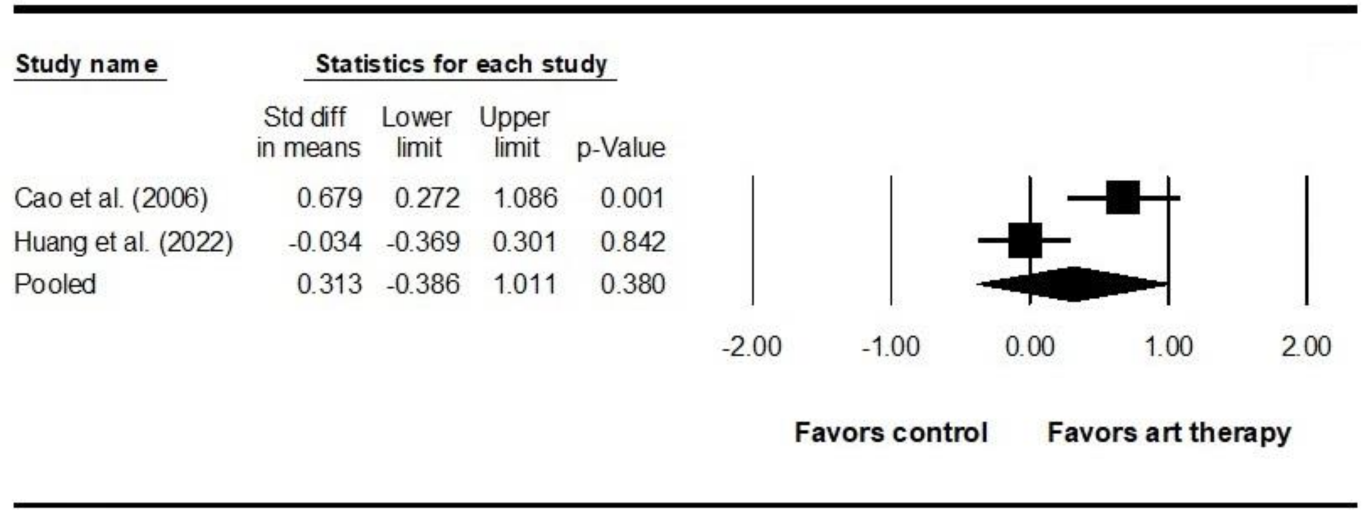

Figure S14. Subgroup analysis of calligraphy for depression.

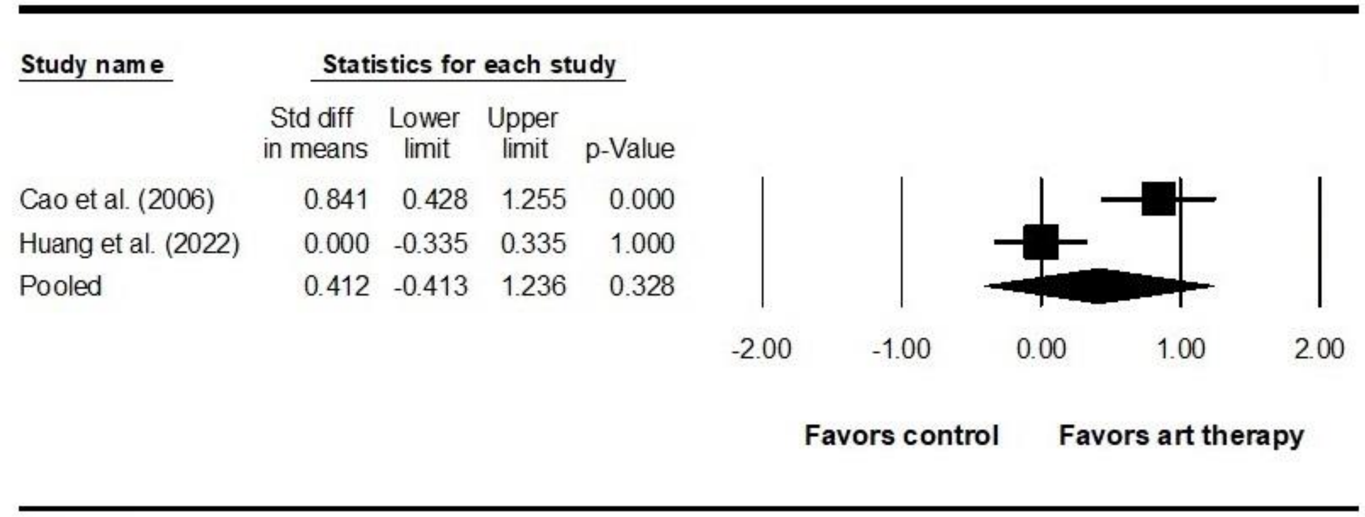

Figure S15. Subgroup analysis of calligraphy for anxiety.

Study name	Std diff in means	Lower limit	Upper limit	p-Value
Dong et al. (2013)	0.207	-0.266	0.680	0.392
Huang et al. (2020)	0.580	0.080	1.080	0.023
Li et al. (2010)	0.869	0.340	1.398	0.001
Zhou et al. (2010)	1.710	1.118	2.301	0.000
Pooled	0.824	0.225	1.422	0.007

(Statistics for each study)

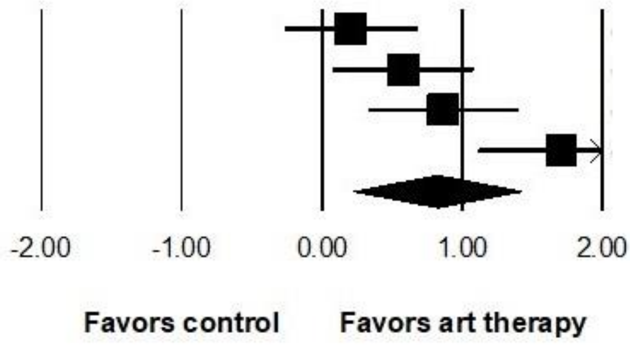

Figure S16. Subgroup analysis of painting and calligraphy for positive symptoms.

Study name	Std diff in means	Lower limit	Upper limit	p-Value
Dong et al. (2013)	0.005	-0.467	0.476	0.985
Huang et al. (2020)	0.025	-0.465	0.515	0.919
Li et al. (2010)	0.961	0.426	1.495	0.000
Zhou et al. (2010)	1.019	0.481	1.557	0.000
Pooled	0.491	-0.059	1.042	0.080

(Statistics for each study)

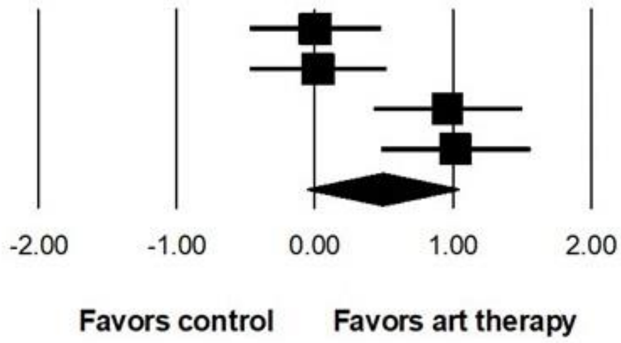

Figure S17. Subgroup analysis of painting and calligraphy for negative symptoms.

Study name	Std diff in means	Lower limit	Upper limit	p-Value
Dong et al. (2013)	0.560	0.079	1.041	0.023
Li et al. (2010)	0.898	0.367	1.428	0.001
Pooled	0.712	0.356	1.069	0.000

(Statistics for each study)

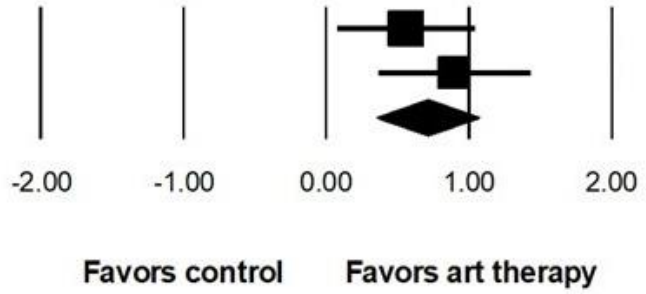

Figure S18. Subgroup analysis of painting and calligraphy for depression.

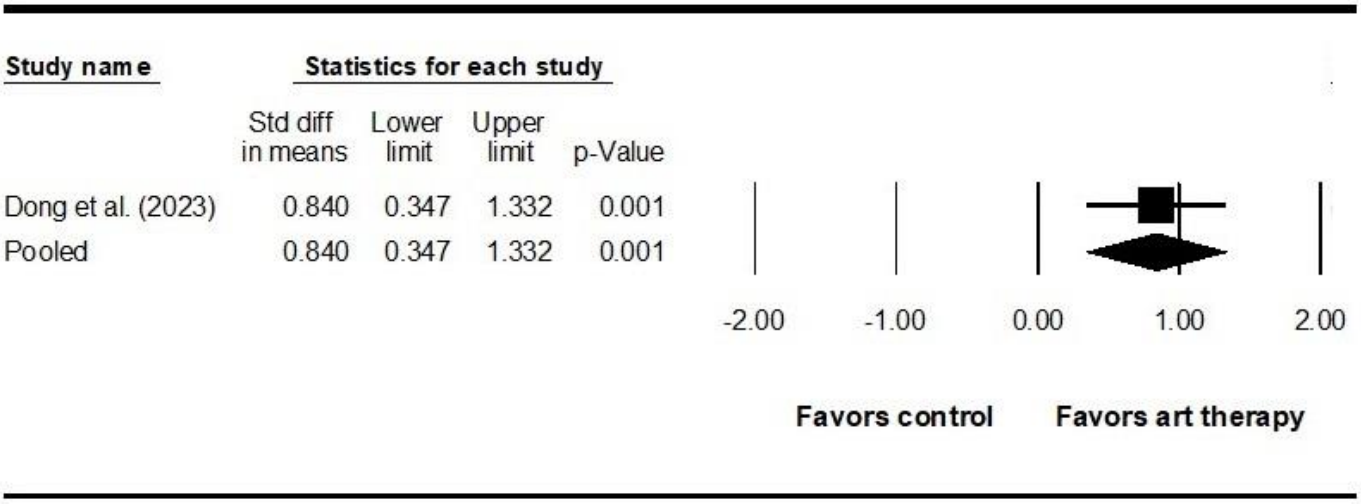

Figure S19. Subgroup analysis of painting and calligraphy for anxiety.

PRISMA 2020 Checklist

Section and Topic	Item #	Checklist item	Location where item is reported
**TITLE**			
Title	1	Identify the report as a systematic review.	Title page
**ABSTRACT**			
Abstract	2	See the PRISMA 2020 for Abstracts checklist.	abstract
**INTRODUCTION**			
Rationale	3	Describe the rationale for the review in the context of existing knowledge.	1-3
Objectives	4	Provide an explicit statement of the objective(s) or question(s) the review addresses.	3
**METHODS**			
Eligibility criteria	5	Specify the inclusion and exclusion criteria for the review and how studies were grouped for the syntheses.	3
Information sources	6	Specify all databases, registers, websites, organisations, reference lists and other sources searched or consulted to identify studies. Specify the date when each source was last searched or consulted.	3
Search strategy	7	Present the full search strategies for all databases, registers and websites, including any filters and limits used.	3, Table S1
Selection process	8	Specify the methods used to decide whether a study met the inclusion criteria of the review, including how many reviewers screened each record and each report retrieved, whether they worked independently, and if applicable, details of automation tools used in the process.	3
Data collection process	9	Specify the methods used to collect data from reports, including how many reviewers collected data from each report, whether they worked independently, any processes for obtaining or confirming data from study investigators, and if applicable, details of automation tools used in the process.	3-4
Data items	10a	List and define all outcomes for which data were sought. Specify whether all results that were compatible with each outcome domain in each study were sought (e.g. for all measures, time points, analyses), and if not, the methods used to decide which results to collect.	3-4
	10b	List and define all other variables for which data were sought (e.g. participant and intervention characteristics, funding sources). Describe any assumptions made about any missing or unclear information.	3-4
Study risk of bias assessment	11	Specify the methods used to assess risk of bias in the included studies, including details of the tool(s) used, how many reviewers assessed each study and whether they worked independently, and if applicable, details of automation tools used in the process.	4
Effect measures	12	Specify for each outcome the effect measure(s) (e.g. risk ratio, mean difference) used in the synthesis or presentation of results.	4
Synthesis methods	13a	Describe the processes used to decide which studies were eligible for each synthesis (e.g. tabulating the study intervention characteristics and comparing against the planned groups for each synthesis (item #5)).	4
	13b	Describe any methods required to prepare the data for presentation or synthesis, such as handling of missing summary statistics, or data conversions.	4
	13c	Describe any methods used to tabulate or visually display results of individual studies and syntheses.	4
	13d	Describe any methods used to synthesize results and provide a rationale for the choice(s). If meta-analysis was performed, describe the model(s), method(s) to identify the presence and extent of statistical heterogeneity, and software package(s) used.	4
	13e	Describe any methods used to explore possible causes of heterogeneity among study results (e.g. subgroup analysis, meta-regression).	4

PRISMA 2020 Checklist

Section and Topic	Item #	Checklist item	Location where item is reported
	13f	Describe any sensitivity analyses conducted to assess robustness of the synthesized results.	4
Reporting bias assessment	14	Describe any methods used to assess risk of bias due to missing results in a synthesis (arising from reporting biases).	4
Certainty assessment	15	Describe any methods used to assess certainty (or confidence) in the body of evidence for an outcome.	4
**RESULTS**			
Study selection	16a	Describe the results of the search and selection process, from the number of records identified in the search to the number of studies included in the review, ideally using a flow diagram.	4-5, Figure 1
	16b	Cite studies that might appear to meet the inclusion criteria, but which were excluded, and explain why they were excluded.	Figure 1
Study characteristics	17	Cite each included study and present its characteristics.	5, Table S2
Risk of bias in studies	18	Present assessments of risk of bias for each included study.	6, Figure 2
Results of individual studies	19	For all outcomes, present, for each study: (a) summary statistics for each group (where appropriate) and (b) an effect estimate and its precision (e.g. confidence/credible interval), ideally using structured tables or plots.	6, Figures 3-6, Table 1
Results of syntheses	20a	For each synthesis, briefly summarise the characteristics and risk of bias among contributing studies.	5-6, Table S2
	20b	Present results of all statistical syntheses conducted. If meta-analysis was done, present for each the summary estimate and its precision (e.g. confidence/credible interval) and measures of statistical heterogeneity. If comparing groups, describe the direction of the effect.	6, Figures 3-6, Table 1
	20c	Present results of all investigations of possible causes of heterogeneity among study results.	7, Table S3
	20d	Present results of all sensitivity analyses conducted to assess the robustness of the synthesized results.	6
Reporting biases	21	Present assessments of risk of bias due to missing results (arising from reporting biases) for each synthesis assessed.	6, Figure 2
Certainty of evidence	22	Present assessments of certainty (or confidence) in the body of evidence for each outcome assessed.	7, Table S4
**DISCUSSION**			
Discussion	23a	Provide a general interpretation of the results in the context of other evidence.	11
	23b	Discuss any limitations of the evidence included in the review.	13
	23c	Discuss any limitations of the review processes used.	13
	23d	Discuss implications of the results for practice, policy, and future research.	11-13
**OTHER INFORMATION**			
Registration and	24a	Provide registration information for the review, including register name and registration number, or state that the review was not registered.	4

PRISMA 2020 Checklist

Section and Topic	Item #	Checklist item	Location where item is reported
protocol	24b	Indicate where the review protocol can be accessed, or state that a protocol was not prepared.	4
	24c	Describe and explain any amendments to information provided at registration or in the protocol.	NA
Support	25	Describe sources of financial or non-financial support for the review, and the role of the funders or sponsors in the review.	13
Competing interests	26	Declare any competing interests of review authors.	13
Availability of data, code and other materials	27	Report which of the following are publicly available and where they can be found: template data collection forms; data extracted from included studies; data used for all analyses; analytic code; any other materials used in the review.	13

From: Page MJ, McKenzie JE, Bossuyt PM, Boutron I, Hoffmann TC, Mulrow CD, et al. The PRISMA 2020 statement: an updated guideline for reporting systematic reviews. BMJ 2021;372:n71. doi: 10.1136/bmj.n71

For more information, visit: http://www.prisma-statement.org/
